# Quantifying the Antioxidant Capacity of Inorganic Nanoparticles: Challenges and Analytical Solutions

**DOI:** 10.3390/antiox14101254

**Published:** 2025-10-18

**Authors:** Yue Hu, Qingbo Zhang, Zhen Xiao, Xiaoting Guo, Vivian Ling, Yidan Bi, Vicki L. Colvin

**Affiliations:** 1 Department of Chemistry, Brown University, Providence, RI 02906, USA; yue_hu@alumni.brown.edu (Y.H.); nanomaterialszh@gmail.com (Q.Z.); zhen_xiao@alumni.brown.edu (Z.X.); xiaoting_guo@alumni.brown.edu (X.G.); vivian_ling@alumni.brown.edu (V.L.); yidan_bi@alumni.brown.edu (Y.B.); 2 School of Engineering, Brown University, Providence, RI 02906, USA

**Keywords:** nanoparticle antioxidants, reactive oxygen species (ROS), antioxidant assays, optical interference correction

## Abstract

Antioxidant properties of inorganic nanoparticles in aqueous media are attracting growing interest due to their high surface reactivity. Materials such as cerium oxide, iron oxide, silver, and gold exhibit distinct radical-scavenging behaviors at the nanoscale, but reliable quantification remains challenging. Conventional assays developed for molecular antioxidants cannot be directly applied because probes such as 2,2-diphenyl-1-picrylhydrazyl (DPPH) require methanol–water mixtures and are unstable in aqueous nanoparticle suspensions, while other assays are affected by nanoparticle-induced absorption or fluorescence changes. Here we demonstrate strategies to correct these interferences by independently measuring nanoparticle optical properties after oxidation and customizing assay conditions to account for the dilute, per-particle concentrations of nanomaterials. Using a high-throughput 96-well format, four adapted assays revealed that silver, ceria, and iron oxide nanoparticles possess substantially higher antioxidant capacities than Trolox, while gold showed negligible activity. This optimized approach enables reproducible comparison of nanoparticle antioxidants and provides a platform for tailoring nanostructures with enhanced radical-scavenging properties.

## 1. Introduction

Nanoparticles often undergo preferential oxidation when exposed to reactive oxygen species (ROS), thereby shielding surrounding molecules from oxidative damage [1,2,3,4]. In most cases, these nanoparticle antioxidants have per-particle antioxidant capacities orders of magnitude larger than that of molecules by virtue of the many oxidation sites present on their surfaces [5,6,7]. This large capacity for oxidation is of great value in diverse applications, ranging from food preservation to biomedicine and biofuels [8,9,10,11]. Nanoparticles also are more thermally stable than antioxidant molecules [12], which makes them ideal for use in extreme environments. Some nanoparticles, such as cerium oxide, show regenerative antioxidant behavior: under physiological conditions, oxidized nanoparticles are slowly reduced by donors and restored to their active form [13,14]. This self-renewing activity provides sustained protection against oxidative stress, a property of particular interest in biological contexts [14,15,16].

Despite hundreds of reports on nanoparticle antioxidant behavior, relatively few studies have quantitatively characterized this property [7,13,17]. This is surprising as studies of molecular antioxidants offer definitive terminology for characterizing oxidation behavior as well as a wealth of standard methods for assessing it [18,19,20]. The success of an antioxidant molecule or nanoparticle antioxidant is characterized by its antioxidant capacity, reflecting the degree of oxidation, as well as the antioxidant reactivity, a measure of the speed with which the antioxidant reacts with reactive oxygen species [21]. Studies quantify these two features of antioxidant behavior using standard antioxidant assays and reporting results against a standard molecule such as Trolox [22,23,24]. Determination of these reactivities has helped investigators optimize the molecular design for antioxidants, as well as illuminate the oxidation mechanisms [25,26]. Later, investigators have adapted antioxidant assays for 96-well formats that permit screening of antioxidant libraries so as to optimize properties better [25,26,27].

Applying these conventional antioxidant characterization methods to nanoparticles presents several challenges. One key limitation is that conventional molecular assays depend on monitoring changes in the absorbance or fluorescence of an indicator molecule (Table 1) [28,29,30,31,32,33]. Nanoparticles, however, can complicate these measurements by quenching fluorescence or altering absorbance spectra during oxidation processes. Similarly, in vitro cytotoxicity assays that rely on optical readouts can be confounded by the complex photophysical properties exhibited by many nanomaterials [34,35,36]. Another challenge lies in the management of the indicator solubility in a colloidal suspension; one of the most popular probes for antioxidant behavior in cell culture, DPPH, is only soluble in alcohol/water mixtures, which are rarely suitable for studying nanoparticles stabilized for physiological conditions [37,38,39]. Finally, many of these assays were originally optimized for detecting antioxidant behavior in millimolar concentrations of molecular antioxidants, oxidants, and indicators, whereas nanoparticle dispersions are often far more dilute, usually on the order of tens to hundreds of nanomolar per particle [40].

In this work, we evaluate four conventional molecular assays of antioxidant properties to quantify the reactivity of different nanoparticle compositions towards oxidation in water. These experiments were geared towards aqueous applications of nanoparticles and utilized organic peroxide (ROO•) or the hydroxyl radical (OH•) as oxidants. Direct application of these molecular protocols to nanoparticle suspensions proved challenging, primarily due to stability issues. For example, the widely used 2,2-diphenyl-1-picrylhydrazyl (DPPH) assay relies on the addition of alcohol to solubilize the hydrophobic indicator, but this condition induces nanoparticle aggregation and precipitation, leading to unreliable measurements. Consequently, assays employing more polar, water-compatible indicators are better suited for nanoparticle systems. In these cases, probe oxidation is monitored via absorbance or fluorescence changes, but optical interference from the nanoparticles themselves must be corrected to obtain accurate measurements. With these corrections, four widely adopted antioxidant assays can be adapted to a 96-well plate setup, enabling systematic evaluation regardless of the inherently low per-particle concentration of nanoparticles.

## 2. Materials and Methods

### 2.1. Materials

Pyrogallol red (PGR), Pyranine (PYR, ≥96%), Rhodamine B (RhB, ≥96%), fluorescein sodium salt (Fluorescein), (±)-6-Hydroxy-2,5,7,8-tetramethylchromane-2-carboxylic acid (Trolox, 97%), L-ascorbic acid (AA, ≥99%), 2,2′-Azobis(2-methylpropionamidine) dihydrochloride (AAPH, granular, 97%), 2,2-diphenyl-1-(2,4,6-trinitrophenyl)-hydrazinyl (DPPH), 2,2′-Azino-bis(3-ethylbenzothiazoline-6-sulfonic acid) diammonium salt (ABTS, ≥98%), hydrogen peroxide (H_2_O_2_, 30 wt. % in H_2_O), phosphate buffered saline (PBS, 10×), gold(III) chloride trihydrate (HAuCl_4_·3H_2_O, ≥99.9%), silver perchlorate (AgClO_4_, 97%), ferric chloride hexahydrate (FeCl_3_·6H_2_O, 97%), cerium(III) nitrate hexahydrate (Ce(NO_3_)_3_·6H_2_O, 99%), sodium nitrate (NaNO_3_, ≥99%), oleylamine (70%), 1-octadecene (90%), oleic acid (≥99%), urea (≥98%), dopamine hydrochloride, (1-Cyano-2-ethoxy-2-oxoethylidenaminooxy)dimethylamino-morpholino-carbenium hexafluorophosphate (COMU, 97%), 4-Methylmorpholine (≥99%), and sodium citrate dihydrate (≥99%) were purchased from Sigma Chemical Co. (St. Louis, MO, USA). Myoglobin from horse heart (≥96%) and phosphate-citrate buffer tab (pH 5) were from the antioxidant assay kit (CS0790) purchased from Sigma Chemical Co. (St. Louis, MO, USA). Poly(ethylene glycol) methyl ether thiol (mPEG-SH, MW 10 K) was purchased from Creative PEGWorks (Durham, NC, USA). 4arm PEG, 3arm-Hydroxyl, 1arm-Acetic Acid (MeO-PEG-COOH, MW 10 K) was purchased from JenKem Technology USA (Plano, TX, USA). 1,2-dioleoyl-sn-glycero-3-phosphoethanolamine-N- [methoxy(polyethylene glycol)-2000] (ammonium salt) (18:1 PEG2000-PE, >99%) was purchased from Avanti Polar Lipids (Alabaster, AL, USA). Polyacrylic acid (PAA, MW 6 KDa) was purchased from PolyScience (Niles, IL, USA). Deionized (DI) water with a resistivity of 18.2 MΩ·cm was used in all experiments.

All solutions were freshly prepared immediately prior to the experiments. Stock solutions of pyranine, rhodamine B, and pyrogallol red (each 500 µM) and sodium fluorescein (400 µM) were prepared in 1× phosphate buffer, while Trolox stock solution (1500 µM) was prepared similarly. Working solutions were obtained by diluting the stocks to final concentrations ranging from 5 to 100 µM. AAPH solutions (10–50 mM) were prepared by dissolving the solid in PBS. Gold, silver, iron oxide, and ceria nanoparticles (0.1–5 nM) were diluted in phosphate buffer immediately before measurement.

### 2.2. Assay Methods

Absorbance and fluorescence measurements on 96-well microplates were performed using a SpectraMax M3 microplate reader (Molecular Devices, San Jose, CA, USA) equipped with spectrophotometric detection. Absorbance measurements in micro-cuvettes were conducted using a Cary 5000 UV-Vis-NIR spectrometer (Agilent, Santa Clara, CA, USA).

#### 2.2.1. The Trolox Equivalent Antioxidant Capacity (TEAC) Assay Using Pyranine, Pyrogallol Red, and Rhodamine B as Indicators

The assays were conducted in 96-well plates using a solution volume of 250 µL per well [29]. In the TEAC-pyranine and TEAC-rhodamine B assays, 100 µL of Trolox or nanoparticle solution was combined with 20 µL of pyranine or rhodamine B and 50 µL of PBS. The mixture solution was then incubated at 37 °C for 15 min. Then, 80 μL AAPH was injected into the mixture, and the resulting mixture was then dispensed into each well using a multichannel pipette (8 channels, adjustable volume of 10–100 uL, Vision Scientific, Westland, MI, USA). After loading with solutions, the plate was moved to the reader within 2 min, where it was maintained at 37 °C for measurement. UV absorbance was recorded every 30 s for 5 h at 454 nm (pyranine) or 555 nm (rhodamine B) at 37 °C, with the plate shaken for 3 s prior to each measurement. Measurements were performed in triplicate, with standard deviations indicating the random error depicted by the error bars. For the TEAC-pyrogallol red assay, the final reaction mixture contained 150 μL of antioxidant solutions, 20 μL of pyrogallol red, 20 μL of AAPH, and 60 μL of PBS. The absorbance for pyrogallol red was collected at 540 nm.

#### 2.2.2. ORAC Assay Employing Sodium Fluorescein (NaFluo) as Indicator

The antioxidant activity of the samples was evaluated following a standard protocol [32]. In a 96-well black plate, 25 µL of Trolox (0–100 ppm) or nanoparticles (0.1–5 nM) was added to each well, followed by 150 µL of sodium fluorescein (40 nM). The plate was incubated at 37 °C for 15 min. Subsequently, 25 µL of AAPH (150 mM) was added using a multichannel pipette to initiate peroxy radical formation. The plate was quickly transferred to a reader maintained at 37 °C, with excitation and emission filters set at 485 nm and 535 nm, respectively. Fluorescence was recorded every 30 s for 3 h, with a 3 s shaking step before each measurement. Nanoparticles were tested at three concentrations, and all measurements were performed in triplicate. Standard deviations were used to represent the random error shown by the error bars.

#### 2.2.3. DPPH Radical Scavenging Assay in Methanol

Twenty-five microliters of antioxidants (Trolox or nanoparticles) was added to 175 μL DPPH methanol solution (0.5 mM) [41]. Mixtures (final volume 200 µL) were added to 96-well plates with low-evaporation lids, and absorbance at 520 nm was recorded every 3 min for 5 h at 25 °C. Measurements were performed in triplicate, with standard deviations representing the random error.

#### 2.2.4. ABTS Assay Using H_2_O_2_/Myoglobin as Oxidants

The method was modified from that described by the Antioxidant Assay Kit CS0790 (Sigma Chemical Co.). The ABTS probe solution was prepared by dissolving 10 mg of ABTS powder in 100 mL of phosphate-citrate buffer (pH 5). Myoglobin solution was made by dissolving 1.00 mg of horse heart myoglobin in 285 µL of deionized water, and the stock was diluted 100-fold with PBS to obtain the working solution. The ABTS working solution was prepared by adding 25 µL of 3% H_2_O_2_ to 10 mL of the ABTS substrate and used within 30 min. Assays were conducted in 96-well plates, where 10 µL of antioxidant (nanoparticles, 0.1–5 nM, or Trolox, 0–100 µM) was added to each well, followed by 20 µL of myoglobin working solution. Subsequently, 150 µL of ABTS working solution was introduced, and the plate was transferred to the reader within 2 min. Absorbance at 415 nm was recorded at room temperature every 30 s for 1 h. Nanoparticles were tested at three concentrations, and all measurements were performed in triplicate. Standard deviations were used to represent the random error shown by the error bars.

#### 2.2.5. Measurement of TROLOX Equivalent Antioxidant Capacity (TEAC)

##### Using the Time-Dependent (Half-Life, t_1/2_) Data to Estimate TEAC

In ORAC assays using sodium fluorescein, pyranine, rhodamine B, and pyrogallol red as probes, the half-life (t_1/2_) of probe consumption was used to compare the uncorrected TEAC of five types of nanoparticles (Au, Ag, Fe_3_O_4_, Ce_x_Oᵧ-PEG-long (10 K), and Ce_x_Oᵧ-PEG-short (5 K)). The half-life was defined as the time required for the probe signal to decrease to 50% of the total change (Signal_initial_ + Signal_terminal_)/2) during oxidation. A calibration curve was constructed by plotting t_1_/_2_ against Trolox concentrations ranging from 0 to 50 µM. The equations of the calibration curves are y = 52.63 x + 259, r^2^ = 0.992 for sodium fluorescein; y = 438.74 x + 1339, r^2^ = 0.999 for pyranine; y = 76.00 x + 7584, r^2^ = 1.000 for rhodamine B; y = 34.56 x + 551.93, r^2^ = 0.964 for pyrogallol red.

To obtain the corrected TEAC for the five types of nanoparticles, the t_1/2_ of a corrected probe consumption curve was compared against the calibration curve. The corrected probe consumption curve was generated by using the original probe consumption curve minus the absorption of nanoparticles oxidized by AAPH.

##### Estimation of TEAC Based on Area-Under-the-Curve (AUC) Analysis [32,42]

Due to the varying oxidation rates of the five types of nanoparticles, TEAC is more accurately determined using the area under the probe consumption curve (AUC). In ORAC assays using sodium fluorescein, pyranine, rhodamine B, and pyrogallol red as probes, the uncorrected TEAC of the five types of nanoparticles was compared using the relative AUC (AUC-AUC_0_) of probe consumption. AUC_0_ is the AUC of Trolox when the concentration equals 0 µM. The AUC and AUC_0_ of the probe consumption curve were calculated by the OriginLab Integrate function with the baseline (Y = Probe signal value at the end of the oxidation process). A calibration curve was constructed by plotting (AUC-AUC_0_) against Trolox concentrations ranging from 0 to 20 µM. The equations of the calibration curves are y = 1361.28 + 7055.77 x, r^2^ = 0.996 for sodium fluorescein; y = 3.92 x + 0.0489, r^2^ = 0.995 for pyranine; y = 3.78 x + 0.0784, r^2^ = 0.991 for rhodamine B; y = 1.25 x + 2.43, r^2^ = 0.972 for pyrogallol red.

To obtain the corrected TEAC for the five types of nanoparticles, the relative AUC of the probe consumption minus the AUC of nanoparticle UV-absorbance during the oxidation process (AUC-AUC_0_-AUC_NP oxidation_) was compared to the calibration curves in ORAC-pyranine, ORAC-rhodamine B, and ORAC-pyrogallol red assays. In the ORAC-fluorescein assay, the relative AUC of the probe consumption minus the AUC of the probe-nanoparticle interaction (AUC-AUC_0_-AUC_probe-NP_) was compared to the calibration curve.

##### Using ABTS Assay to Estimate TEAC [43,44]

Absorbance (λ = 415 nm) at 5 min after the injection of H_2_O_2_/myoglobin was used to calculate TEAC. Absorbance was plotted vs. nanoparticle concentrations (Figure S14). The TEAC was obtained by using the equation TEAC(µM Trolox/µM NP) = slope_NP_/slope_calibration curve_. The slope of the Trolox calibration curve (slope_calibration curve_) was obtained by plotting abs (λ = 415 nm) at 5 min vs. Trolox concentrations, with the fitted equation y = −0.00374 x + 0.980, r^2^ = 0.894.

#### 2.2.6. Ultraviolet–Visible (UV) Absorption Spectra of Oxidized Nanoparticles

Absorption spectra of nanoparticle solutions (5–100 ppm) were recorded from 350 to 800 nm using a Cary 5000 UV–Vis–NIR spectrophotometer (Agilent, USA). To assess nanoparticle oxidation, 200 µL of nanoparticle solution was combined with 140 µL of PBS in a disposable polystyrene micro-cuvette with a 10 mm path length and incubated at 37 °C for 5 min. Subsequently, 160 µL of AAPH (50 mM in PBS) was added, and absorption spectra from 350 to 800 nm were recorded every 10 min during the first 30 min and every 20 min over the following 2 h.

### 2.3. Nanoparticle Preparation

#### 2.3.1. Synthesis of 4.7 nm Ceria Nanoparticles, Concentration Determination, and Phase Transfer into Water

Ceria nanoparticles were synthesized through high-temperature decomposition of ceria carboxylates [45]. Briefly, one mmol of Ce(NO_3_)_3_·6H_2_O, 3.0 mmol of oleylamine, and 4.0 g of 1-octadecene were combined in a 50 mL three-necked glass flask. The right neck held a Digi-Sense thermocouple high-temperature probe in conjunction with a Digi-Sense standard temperature controller to provide precise temperature control of the process. The middle neck was connected to the Argon tank, thus providing a stable argon flow. The left neck was connected to a rotovap bump trap (100 mL, ChemGlass, Vineland, NJ, USA), aiding condensation of low-boiling-point impurities during the reaction. Then, the trap was connected to a glass oil bubbler filled with silicone oil to control the argon gas flow by observing the speed of bubble (2 bubbles per second) generation. The flask was purged with argon for 10 min and then heated to 80 °C for 30 min, yielding a brownish-yellow mixture. The temperature was raised to 120 °C for 30 min to remove low-boiling impurities, followed by heating to 260 °C for 2 h. The final product was precipitated using acetone (Sigma-Aldrich, St. Louis, MO, USA, >99.5%) and methanol (Sigma-Aldrich, St. Louis, MO, USA, ≥99.85%). The sediment was collected by centrifugation, then redispersed into hexanes (>98.5%) or CHCl_3_ (Sigma-Aldrich, St. Louis, MO, USA, >99%). This procedure was repeated at least five times until the sample no longer exhibited a waxy texture.

Ceria nanoparticles were transferred from these solutions into deionized water via a phase transfer process using 10K-branched NitroDOPA-PEG polymer or 1,2-dioleoyl-sn-glycero-3-phosphoethanolamine-N-[methoxy(polyethylene glycol)-2000] (18:1 PEG-2000-PE). Briefly, 15 mg PEG (MW = 2000) was dissolved in 1.0 mL of CHCl_3_, then 0.5 mL of ceria nanoparticles (0.5 mL, 2000 ppm Ce) was added. The resulting mixture was stirred vigorously for 20 min, then 3mL DI water was added, followed by an additional day of vigorous stirring under ambient conditions in a chemical hood. During this time, the CHCl_3_ evaporated, leaving an aqueous suspension of ceria nanoparticles that appeared brownish-yellow. The final product was purified first by passage through a 0.2-micron syringe filter, followed by successive centrifugation cycles. In a typical procedure, the solution was placed in a centrifugal filter (100 KDa, Pall) and subjected to centrifugation (3000 rpm, 4481× *g*, 15 min), leaving a solution of particles free from smaller molecular and polymeric impurities. Typical phase transfer yields exceeded 98%.

#### 2.3.2. Nanoparticle Concentrations Were Measured Using Inductively Coupled Plasma Atomic Emission Spectroscopy (ICP-AES)

Twenty microliters of ceria nanoparticle solution was added to 1 mL of HNO_3_ and 0.5 mL of 30% H_2_O_2_, and the mixture was transferred into Teflon digestion vials. The vials were placed in an ultraWAVE Single Reaction Chamber (SRC) Microwave Digestion system (Milestone, Shelton, CT, USA), which was pressurized with 40 bar of argon. Microwave digestion was carried out by ramping the temperature to 240 °C over 20 min, holding for 10 min, and then cooling to room temperature over 20 min, during which the ceria nanoparticles dissolved. The resulting solution was diluted to 10 mL and analyzed using an iCAP 7400 ICP-OES (Thermo Scientific, Waltham, MA, USA). Cerium concentrations were determined by comparison with a calibration curve prepared from cerium standards (1000 mg/L Ce in HNO_3_, Sigma-Aldrich, St. Louis, MO, USA). The original cerium concentration was calculated by applying the appropriate dilution factor.

The nanoparticle molarity was then calculated using the following equation:

(1) $${Molarity}_{NP}=\frac{C_{element}}{w_{element}\%\times \rho \times V_{NP}\times N_{A}}$$

$C_{element}—Elemental\;concentration\;of\;cerium\;w_{element}\%—The\;weight\;percent\;of\;Ce\;in\;CeO_{2}\;\rho —Density\;of\;CeO_{2}\;V_{NP}—Volume\;of\;per\;nanoparticle\;N_{A}—Avogadro^{’}s\;number$

#### 2.3.3. Synthesis of 10 kDa-Branched DOPA-PEG

A custom polymer was synthesized following an existing protocol [46]. One gram of dopamine was dissolved in 30 mL of deionized water, and 1.3 g of sodium nitrate was added to the solution under vigorous stirring in an ice bath. Subsequently, 10 mL of 20% (*v*/*v*) sulfuric acid was added dropwise. After removing the ice bath, the mixture was stirred at room temperature for at least 12 h. The product, nitrodopamine hydrogensulfate (nitroDOPA), was isolated by filtration and washed several times with cold deionized water. The solid was then freeze-dried using a FreeZone 6 Liter Freeze Dry System (LABCONCO, Kansas City, MO, USA) for 2 days. The dry powder was stored in the refrigerator under ambient conditions. For DOPA-PEG synthesis, 0.5 mmol of MeO-PEG-COOH (4arm PEG, 3arm-hydroxyl, 1arm-acetic acid, MW 10 kDa) was dissolved in 10 mL DMF at 37 °C and then cooled to 4 °C in an ice bath. Separately, 0.6 mmol of COMU [(1-Cyano-2-ethoxy-2-oxoethylidenaminooxy)dimethylamino-morpholino-carbenium hexafluorophosphate] was dissolved in 2 mL DMF and combined with 6 mmol N-methylmorpholine, stirring at 4 °C for 20 min. A solution of nitrodopamine (0.6 mmol) in 2 mL DMF was added dropwise, and the reaction proceeded at 4 °C for 2 h, followed by 16 h at room temperature. The polymer was dialyzed against DI water for 48 h and collected by freeze-drying. ^1^H-NMR spectra were recorded on a Bruker 600 MHz NMR using CDCl_3_ (99.5%, Cambridge Isotope Laboratories, Tewksbury, MA, USA).

#### 2.3.4. Synthesis of 17.7 nm Gold Nanoparticles, Concentration Determination, and Phase Transfer

An aqueous solution of HAuCl_4_·3H_2_O (1 mmol in 10 mL) was heated to boiling, and a preheated 10% (*w*/*w*) sodium citrate solution was added at a ratio of 1 mL per 10 mL of gold solution. The mixture was then boiled for 15 min, during which citrate reduced the gold ions, forming gold nanoparticles. As nucleation and growth proceeded, the solution changed color sequentially from yellow to colorless, then gray, purple, and finally dark red. After 15 min, the solution was rapidly cooled in room temperature water, yielding highly crystalline gold nanoparticles with sizes of 10–15 nm [47]. Gold nanoparticles were functionalized using PEG-SH with a molecular weight of 10 kDa [48]. An excess of PEG-thiol (~3000 PEG molecules per particle) was added to the gold nanoparticle solution. The mixture was stirred for at least 12 h to allow the polymer coating to form and stabilize. Unreacted PEG-thiol was removed by centrifugation using a 100 kDa centrifugal filter (Pall) three times at 4150 rpm (6199× *g*) for 15 min. The remaining ~1 mL of pellet was resuspended in DI water. The concentration of the purified solution was then adjusted to maintain a consistent optical density at the peak absorbance (~525 nm). For 17.7 nm gold nanoparticles, the solution was diluted to achieve an optical density corresponding to 21.3 nM. Concentrations were determined based on the absorption coefficients reported by Liu et al. [49].

#### 2.3.5. Synthesis of PAA-Coated 40 nm Iron Oxide Nanocluster and Concentration Determination

The multicore iron oxide nanoparticle clusters were synthesized through a hydrothermal method [50]. In a typical synthesis, 540 mg FeCl_3_·6H_2_O is first dissolved in 20 mL ethylene glycol, followed by 250 mg PAA, 1200 mg urea, and 2000 mg DI water. The mixture was transferred to an autoclave and heated at 185 °C for 6 h. Transmission electron microscopy (TEM) analysis showed that the nanocrystals had an average cluster size of 40.0 ± 3.0 nm and a primary particle size of 4.0 ± 0.5 nm. These materials are coated with poly-acrylic acid and are stable in water.

The concentration of the iron oxide cluster solution was measured using UV–Vis absorption spectroscopy (Cary 5000, Agilent, Santa Clara, CA, USA) [51]. A calibration curve for spectrophotometric analysis of iron was first obtained from iron standards. The standard curve was obtained by mixing 0.2 mL iron (Fe(NO_3_)_3_) standard solution (1, 5, 10, 25, and 50 ppm, respectively, in terms of Fe), 0.15 mL 7.5 M ammonium acetate solution, 0.25 mL 5% hydroxylamine hydrochloride solution, 0.4 mL 0.1% ferrozine solution, and 1 mL H_2_O and placed in a 4 mL quartz cuvette. The absorption peak of Fe(III)-ferrozine composite was at 590 nm in agreement with the existing literature, and the peak intensity was linear with the concentration of iron. To determine the concentration of the cluster solution, 0.1 mL of the sample was dissolved in 0.89 mL of 37% hydrochloric acid with 0.01 mL H_2_O_2_, resulting in a clear, pale-yellow solution. A 0.1 mL aliquot of this solution was then diluted with 0.9 mL H_2_O. Finally, 0.2 mL of the diluted solution was mixed with 0.15 mL of 7.5 M ammonium acetate, 0.25 mL of 5% hydroxylamine hydrochloride, 0.4 mL of 0.1% ferrozine, and 1 mL of H_2_O in a 4 mL quartz cuvette. The absorption spectrum was collected and compared to the standard curve. The measured concentration, in terms of ppm iron, was multiplied by a factor of 1.38 (molar mass ratio between Fe and 1/3 Fe_3_O_4_) to express the concentration in terms of ppm Fe_3_O_4_ for the original cluster solution.

#### 2.3.6. Synthesis of Silver Nanoparticles with a 4.7 nm Diameter, Including Concentration Measurement and Phase Transfer

AgClO_4_ (689 mg) was combined with 45 mL 1-octadecene and 6 mL oleic acid in a 100 mL round-bottom flask connected to a Schlenk line. The mixture was purged with argon and subjected to vacuum three times to remove oxygen, then heated to 70 °C for 30 min. Subsequently, 0.36 mL oleylamine was rapidly injected into the clear solution. The reaction was continued at 150 °C for 4 h, yielding a dark brown solution. The as-synthesized silver nanoparticles were precipitated with ethanol, collected by centrifugation (9000 rpm, 13,444× *g*, 15 min), and redispersed in hexane. This washing procedure was repeated at least five times. The purified nanoparticles were suspended in hexane and stored under nitrogen in a dark vial.

For phase transfer to water, the hexane nanoparticles were precipitated with ethanol, collected by centrifugation (9000 rpm, 13,444× *g*, 10 min), and redispersed in chloroform. Approximately 2.0 mL of nanoparticle solution (~1000 ppm Ag) in chloroform was mixed with 1.0 mL PEG-SH (10 kDa) in chloroform under magnetic stirring. After ~20 min, the top layer turned yellow, indicating successful transfer to the aqueous phase. The aqueous layer was collected through a 0.2 μm syringe filter to remove large precipitates and further purified three times using a 100 kDa centrifugal filter (3000 rpm, 4481× *g*, 5 min). The purified nanoparticles were redispersed in DI water and stored under argon in a dark vial at refrigerated conditions. Silver nanoparticle concentrations were determined by ICP-AES. Briefly, 20 μL of nanoparticle solution was dissolved in 1 mL 50% HNO_3_, diluted to 10 mL, and analyzed using an iCAP 7400 ICP-OES (Thermo Scientific). Concentrations were obtained by comparison with a calibration curve prepared from silver standards (1000 µg/mL, Inorganic Ventures, Christiansburg, VA, USA). The original nanoparticle concentration was calculated by applying the dilution factor, and the molarity was determined using the same equation applied for cerium nanoparticles (Equation (1)).

### 2.4. Nanoparticle Characterization

#### 2.4.1. Transmission Electron Microscopy (TEM)

TEM images were obtained using a JEOL 2100 Field Emission Gun Transmission Electron Microscope (Akishima, Japan) operated at 200 kV. Five microliters of nanoparticle solution (500 ppm) was dropped onto a carbon-coated copper grid (200 mesh, Ted Pella, Redding, CA, USA). After the solvent completely evaporated, the nanoparticles remained on the grid for imaging. Particle diameters were analyzed using ImageJ (v1.54g), and the average size and size distribution were calculated from measurements of at least 1000 nanoparticles.

#### 2.4.2. Dynamic Light Scattering (DLS)

The hydrodynamic radius of nanoparticles in PBS and 50% (*v*/*v*) methanol was measured using a Zetasizer Nano ZS (Malvern Instruments Ltd., Malvern, UK). A 1.5 mL sample of nanoparticles (100 ppm) was placed in a disposable cuvette with a 10 mm pathlength. After allowing 2 min for equilibration, DLS measurements were performed at room temperature. Each sample was measured in triplicate.

## 3. Results

**Scheme 1:**
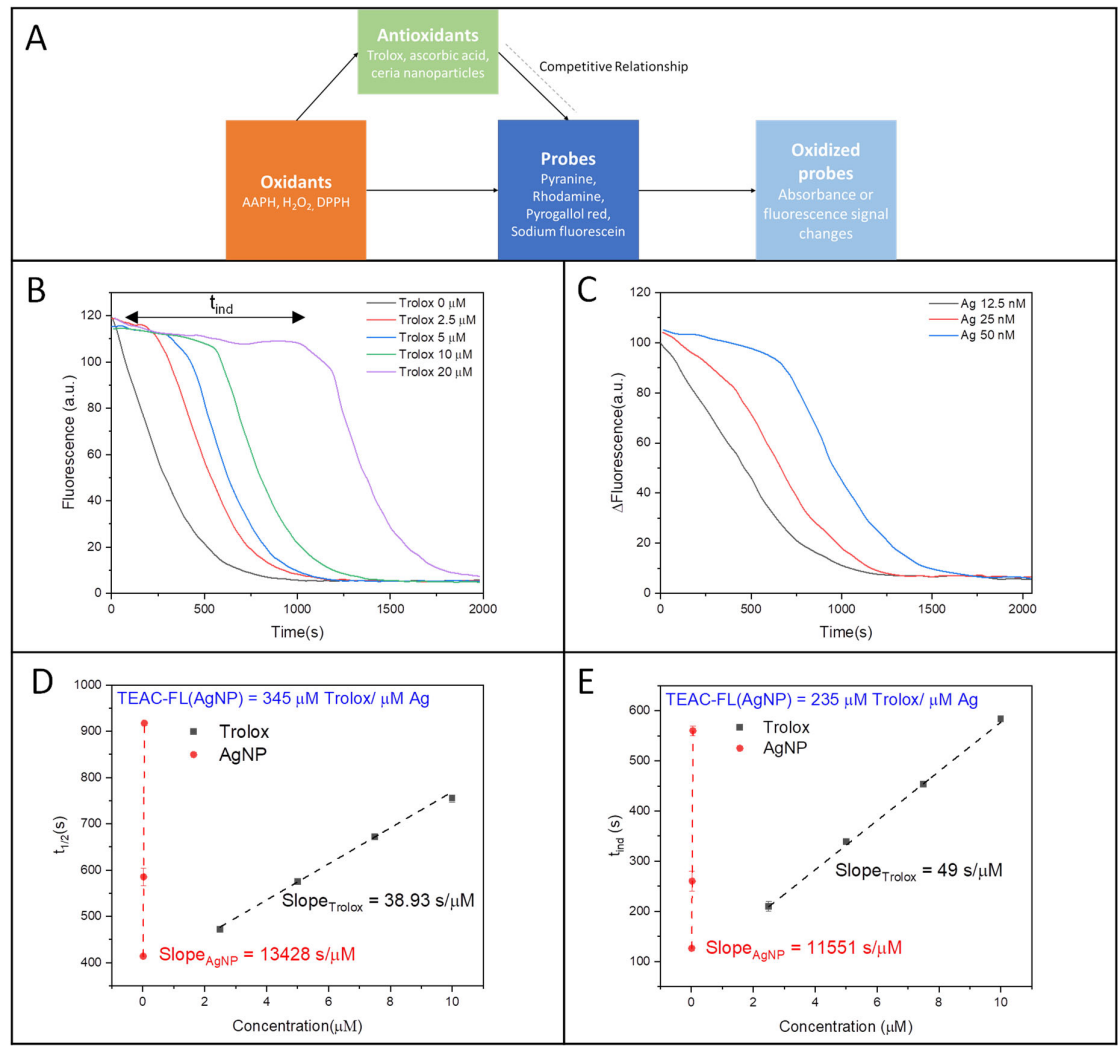
Mechanism of antioxidant assays: (**A**) Measurement of Trolox equivalent antioxidant capacity (TEAC) is found by measuring how a putative antioxidant (green) delays the oxidation of a probe molecule (blue) by a strong oxidant (orange). The most common oxidants are organic peroxy radicals generated when azo compounds undergo thermolysis and hydroxyl radicals formed by transition metal ions (Fe^2+^, Cu^+^) undergoing Fenton chemistry. Probes are selected for their optical properties and how these properties, either ultraviolet–visible absorbance or fluorescence, change after oxidation. (**B**) The oxidation of sodium fluorescein by peroxy radicals, generated from the thermal decomposition of AAPH (2,2′-Azobis(2-amidinopropane) dihydrochloride), is progressively delayed with increasing concentrations of Trolox. In this case, the induction time, indicated by an arrow in the 20 micromolar treatment case, is quantified and correlated with the concentration of antioxidants. (**C**) Similarly, silver nanoparticles can also slow the oxidation of sodium fluorescein, but in this case, the speed of the silver nanoparticle oxidation is comparable to the speed of fluorescein oxidation. In this case, the half-life time (*t*_1/2_) of sodium fluorescein decay is quantified. Overall antioxidant capacity is found by plotting the change in the induction time (**D**) or half-life (**E**) versus the concentration of test antioxidant; effective assays rely on a linear dependence, and the extracted slope is reported relative to a Trolox standard, giving the final Trolox-equivalent antioxidant capacity, followed by a dash indicating the test.

Simple and rapid approaches for assessing antioxidant properties are critical for optimizing molecules and materials for diverse applications. As depicted in Scheme 1A, these assays rely on the oxidation of a molecular probe (blue boxes), which exhibits predictable changes in optical properties—either fluorescence or absorbance—upon oxidation. Reactive oxygen species (ROS), such as hydroxyl radicals (OH•) or organic peroxyl radicals (ROO•), serve as oxidants (orange box). Among these, AAPH is widely used due to its reliable generation of ROO• at elevated temperatures (T > 37 °C). The antioxidant (green box) intercepts these radicals, altering the kinetics of probe oxidation in a measurable way.

When antioxidants exhibit higher reactivity toward oxidants than the probe, they competitively consume the oxidants, thereby preserving the probe and generating an induction period (*t*_ind_, Scheme 1B). Conversely, when the oxidation kinetics of the antioxidant are comparable to those of the probe, the probe’s complete oxidation is delayed, which can be quantitatively assessed by its oxidation half-life (*t*_1/2_, Scheme 1C). Whether investigators used induction time or half-life to evaluate the antioxidant capacities of molecules has generally depended on the form of the time-dependent optical properties. Recent studies on the international standardization of these assays recommend using the area under the curve (AUC) as the preferred metric, since it captures the overall response for any time-dependent measurements [42,52]. Antioxidant capacity was assessed by performing time-dependent measurements at different concentrations of the antioxidant: more antioxidant leads to longer induction times or half-lives, and a linear relationship between antioxidant concentration and AUC can be defined (Scheme 1D). In practice, antioxidant activity is often benchmarked against a reference compound. Trolox, a water-soluble vitamin E analog (6-hydroxy-2,5,7,8-tetramethylchroman-2-carboxylic acid), is the most commonly used standard. Capacities are then expressed as Trolox Equivalents, meaning that a compound with a Trolox Equivalence of one produces the same protective effect on probe oxidation as Trolox at an equivalent molar concentration. Terminology in the literature is not entirely uniform: some studies describe these outcomes as Oxygen Radical Absorbance Capacity (ORAC), whereas others report them as Trolox Equivalent Antioxidant Capacity (TEAC) [42,53].

Although the general framework of optical readout-based antioxidant assays is conceptually universal, the specific experimental implementations vary considerably with respect to the radical generators and reporter probes employed for both small-molecule antioxidants and nanoparticle systems (Table 1). Among the most frequently utilized oxidants is AAPH, which undergoes thermal decomposition at 37 °C to yield alkylperoxyl radicals (ROO•), with a characteristic half-life of approximately 100 s. Other common oxidizing agents include hydrogen peroxide, either alone or in combination with transition metals such as iron, to produce hydroxyl radicals •OH. A unique example is 2,2-diphenyl-1-picrylhydrazyl (DPPH), in which the radical itself serves as the detection probe. Additional probes described in Table 1 are advantageous due to their aqueous stability and the predictable alterations in their optical signatures upon oxidation. Typically, these alterations manifest as decreases in absorbance; however, ABTS (2,2′-azino-bis(3-ethylbenzthiazoline-6-sulfonic acid)) represents a notable exception, since its oxidized form exhibits enhanced absorption in the red region. Fluorescein sodium (sodium 3-oxo-3H-spiro [isobenzofuran-1,9′-xanthene]-3′,6′-bisolate) remains the most widely adopted fluorescence probe, with applications spanning both solution-phase and cellular assays. The relative popularity of these assays for evaluating antioxidant properties was examined through keyword searches in the Web of Science database. These six assays have recently attracted considerable attention for characterizing the antioxidative behavior of both small molecules and nanoparticles [4].

**Figure 1:**
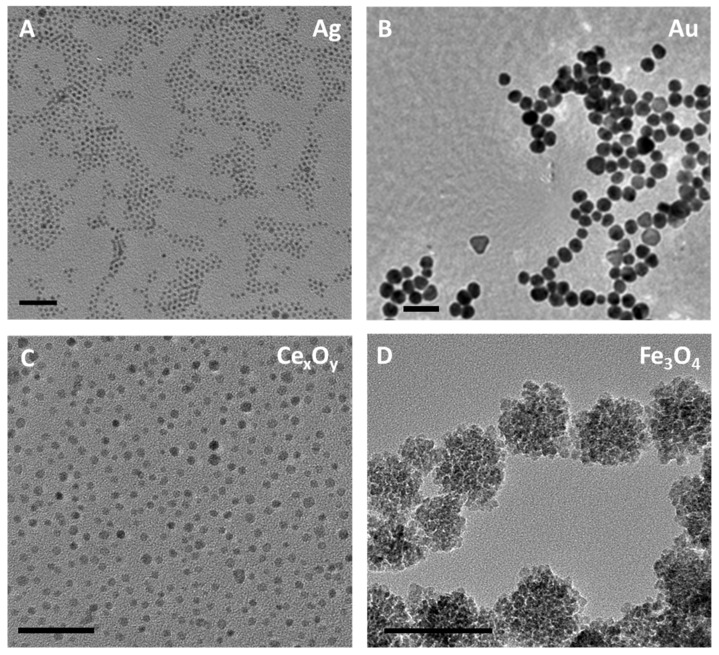
Transmission electron micrographs (TEM) of nanoparticles used in antioxidant capacity measurements: (**A**) Silver nanoparticles (4.5 ± 0.6 nm diameter) coated with oleic acid prior to phase transfer into the water. (**B**) Gold nanoparticles (17.7 ± 3.3 nm diameter) coated with trisodium citrate. (**C**) Ceria nanoparticles (4.7 ± 1.0 nm diameter) coated with oleylamine prior to phase transfer. (**D**) Iron oxide nanoclusters (40.0 ± 3.0 nm diameter, primary crystallites of 4.0 ± 0.5 nm diameter) coated with polyacrylic acid. Several hundred particles were measured in each case to arrive at the average diameters reported, with the standard deviation of the particle dimensions reported as the ± spread. Scale bars are 50 nm.

To examine the relevance of conventional assays to nanoscale systems, we selected four representative antioxidant nanomaterials (Figure 1). As is apparent in electron microscopy, these materials are of nanoscale dimensions, and for these tests, they were suspended in water. Reports of antioxidant activity in silver nanoparticles date back to 2007, where this property was linked to oxidative dissolution of silver. Comparable processes were later proposed for gold nanoparticles [54,55,56]. Iron oxide nanoparticles, containing surface-exposed Fe(II), are also capable of reducing oxidants [57,58]. Among these materials, cerium oxide nanocrystals stand out as a particularly potent antioxidant, first recognized in aqueous media in 2014 [59]. Their distinctive feature is the ability to cycle between oxidized and reduced states, thereby regenerating their active form [13,60]. To ensure colloidal stability in aqueous suspension, nanoparticles were coated with polymers, and atomic analyses were used to determine elemental and per-atom concentrations (Supplemental Table S1). A critical point for applying these assays to nanomaterials is that nanoparticle dispersions are usually far more dilute in terms of particle number compared with the concentrations of probes and oxidants used in the assays.

**Figure 2:**
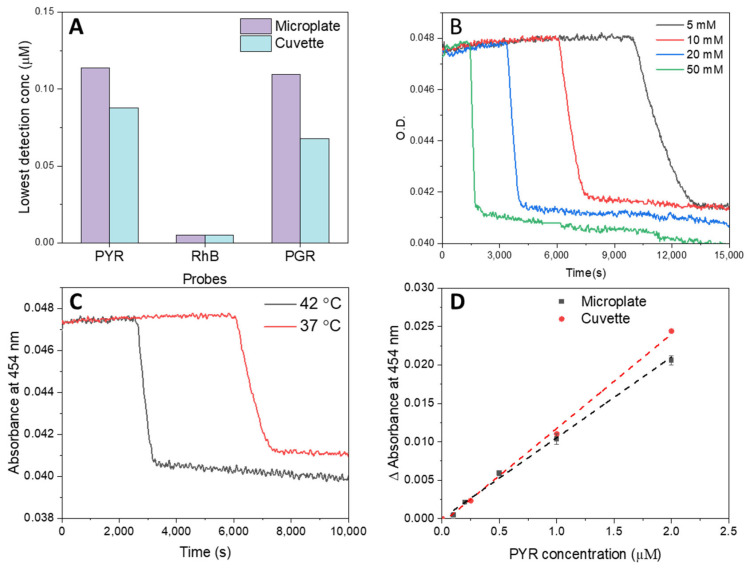
Probe performance under different conditions: (**A**) Minimum detectable concentration of probes in a 96-well plate compared with a conventional cuvette using a high-performance optical absorbance spectrometer. Limits of detection (LOD) are shown for pyranine, Rhodamine B, and pyrogallol red in a 96-well microplate (purple, path length ≈ 8 mm, volume = 250 µL) and in a cuvette (blue, path length = 10 mm). (**B**) Oxidation of pyranine (10 µM, 20 µL) induced by AAPH decomposition at 37 °C, with Trolox protection (10 µM, 100 µL). (**C**) Oxidation of pyranine (10 µM, 20 µL) induced by AAPH decomposition at 37 °C (red) and 42 °C (black), with Trolox protection (10 µM, 100 µL). (**D**) Comparison of assay performance in a 96-well plate versus a standard cuvette and scanning absorption spectrometer. Within experimental error, the slopes are statistically indistinguishable (*p* < 0.001).

For biomedical and environmental studies involving nanoparticles, aqueous suspensions are typically prepared at atomic concentrations ranging from 10 to 100 ppm (mg atom/L), which correspond to particle number concentrations of approximately 10–100 nM. These concentrations are several orders of magnitude lower than the reagent levels conventionally optimized for molecular redox assays, such as probes and oxidants. Lowering the concentrations of both probes and antioxidants to match the dilute nanoparticle suspensions is not generally successful. As illustrated in Figure 2, absorbance-based probes cannot be diluted as the detection of the oxidation can become compromised at concentrations below 0.13 µM for most probes, especially in the short pathlengths of the 96-well plate formats (Figure 2A). Although detection at lower probe concentrations is feasible using highly sensitive spectrophotometers, this approach limits throughput (Figure 2D). Similarly, reducing oxidant concentrations to better align with nanoparticle levels is problematic, as the resulting slower reaction kinetics complicate assay interpretation (Figure 2B). Increasing the temperature of the AAPH decomposition, however, can yield greater reactive oxygen species even under lower AAPH concentration, leading to faster reactions (Figure 2C). Within these constraints, it remains preferable to minimize oxidant and probe levels as much as possible so that they approximate the nanomolar concentrations of nanoparticle antioxidants. Quantitative comparisons provided in Table 2 highlight the influence of probe and oxidant concentrations on the oxidation response. Notably, across a wide range of experimental conditions, the relative antioxidant activity of a model cerium oxide nanoparticle, measured using the pyranine-based ORAC-PYR assay, remains consistent.

**Figure 3:**
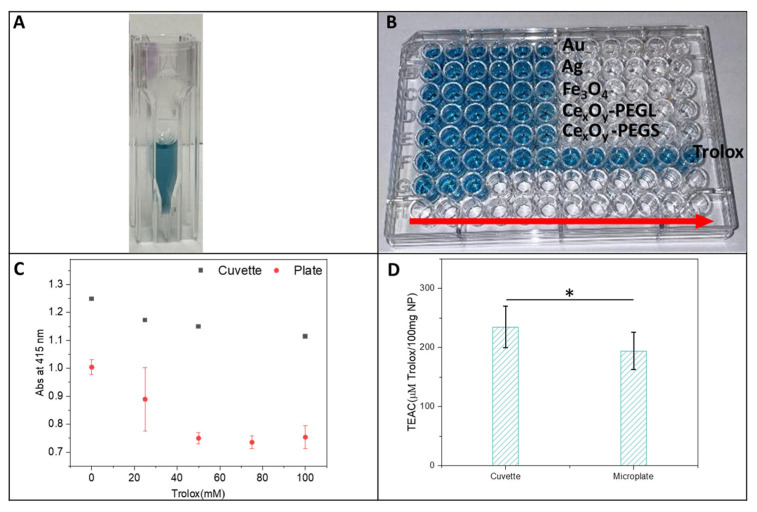
Photographs of TEAC-ABTS assays executed in a cuvette (**A**) and 96-well plate (**B**). The red arrow to the right indicates increasing concentrations of Trolox or nanoparticles. Upon oxidation, the probe molecule exhibits an increase in absorbance; therefore, the blue wells on the left correspond to low antioxidant concentrations and pronounced oxidation. (**C**) Absorbance of ABTS•^+^ at 415 nm increases in the presence of H_2_O_2_/myoglobin. As the Trolox concentration increases, the absorbance decreases in both the optical spectrometer (black) and the plate reader (red). (**D**) Trolox equivalent antioxidant capacity (TEAC) of Ce_x_O_y_-PEG 10 K, determined using both a scanning absorption spectrometer and a microplate reader. Error bars represent triplicate measurements. Measurements from the microplate reader yielded TEAC values significantly lower than the cuvette-based assay (* *p* < 0.05).

Recent advances in the antioxidant molecular analysis have highlighted the value of high-throughput platforms enabled by 96-well plates and plate readers (Figure 3) [29,61]. While plate readers generally exhibit lower sensitivity and noisier optical signals compared to conventional spectrometers, these limitations can be mitigated by the ability to perform a large number of replicates. These principles can be applied to nanoparticle assays as well. We compared the ABTS assay performed in a conventional optical spectrometer using a standard 1 cm cuvette (Figure 3A) to those conducted in a 96-well plate format analyzed with a plate reader (Figure 3B). A complete dataset on all our nanoparticles could be collected in a matter of hours in the high-throughput setup (Figure 3B) as opposed to several days in the conventional system (Figure 3A). The primary adjustment involves accounting for the shorter optical pathlength of the plate reader (8 mm at 250 µL) relative to the standard cuvette (10 mm), which necessitates higher probe concentrations. Despite this difference, the Trolox-equivalent reactivity obtained from both methods showed strong consistency, confirming that antioxidant measurements of nanoparticles can be reliably adapted to the 96-well plate format (Figure 3D).

**Figure 4:**
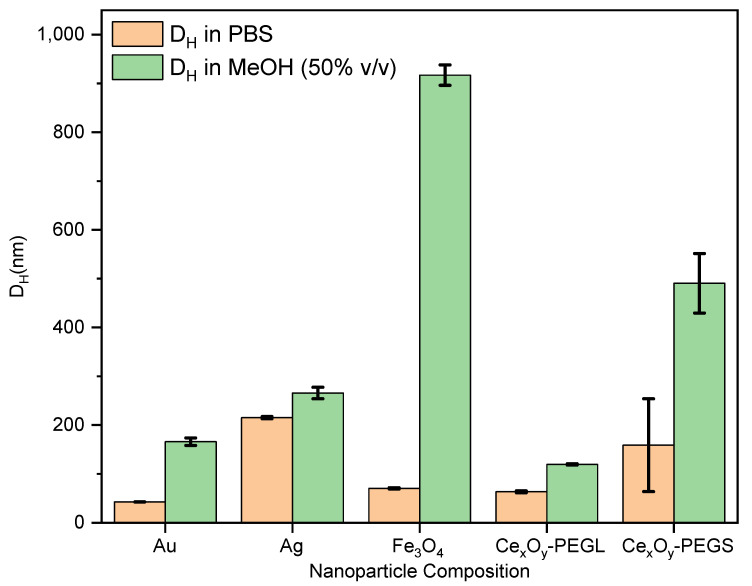
Hydrodynamic diameter (D_H_) of nanoparticles measured in aqueous phosphate-buffered saline and in a 50% *v*/*v* methanol–water mixture, demonstrating the size variation in a less polar medium. In all cases, the change in D_H_ is significant (*p* > 0.01), and progressive sedimentation of the nanoparticles is observed over time.

The six tests listed in Table 1 were used for evaluating the antioxidant capacity of nanoparticles with probe and oxidant concentrations adjusted specifically for nanoparticle testing (Table 2). The DPPH assay, although widely used for molecular antioxidants, proved unsuitable in this context because it relies on co-solvents such as THF or methanol to overcome the poor aqueous solubility of DPPH. These co-solvents are incompatible with nanoparticles, as their reduced polarity frequently causes nanoparticle aggregation and even sedimentation (Figure 4). In such cases, the lack of a stable and homogeneous dispersion results in unreliable data, since optical scattering from aggregates may mimic or mask changes in probe absorbance unrelated to redox processes.

**Figure 5:**
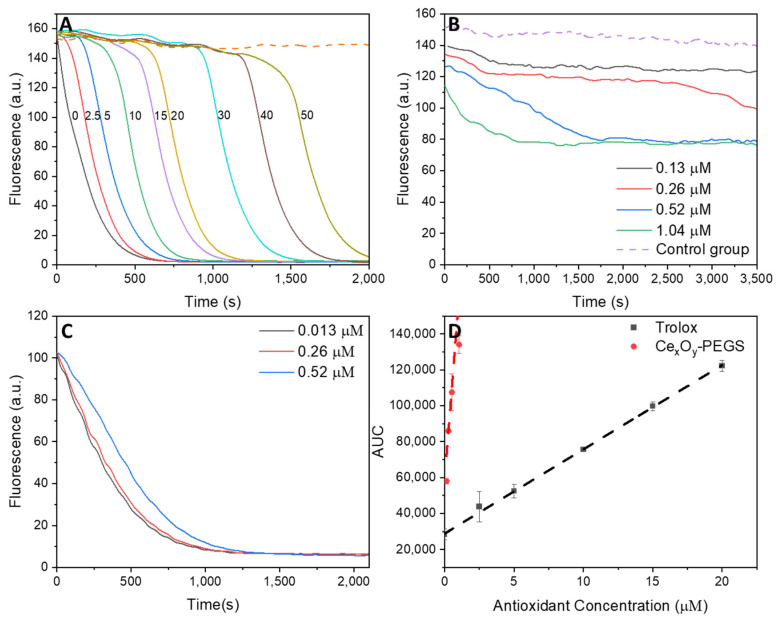
Antioxidant assay using sodium fluorescein as probe: (**A**) Oxidation of sodium fluorescein triggered by AAPH decomposition at 37 °C is progressively delayed with increasing Trolox concentrations (0-50 µM). The red dashed line indicates that fluorescein fluorescence remains unchanged in the presence of Trolox alone (10 µM) without oxidant. (**B**) Introduction of nanoparticles alters the emission profile of sodium fluorescein. Even at low concentrations (0.13-1.04 µM, red dashed line), nanoparticles caused fluorescence quenching, which became more pronounced at higher concentrations (black values). (**C**) Fluorescein oxidation can be separated from nanoparticle-induced quenching by correcting the time-resolved fluorescence data to remove the contribution of nanoparticle–probe interactions. Data shown are for Ce_x_O_y_-PEG-short nanoparticles across multiple concentrations. The progression of oxidation was quantified using the area under the curve (AUC). (**D**) Concentration-dependent changes in AUC for both Trolox and CexOy-PEG-short nanoparticles after applying the correction for nanoparticle–probe interactions. Without this adjustment, the antioxidant capacity of the nanoparticles is overestimated.

**Figure 6:**
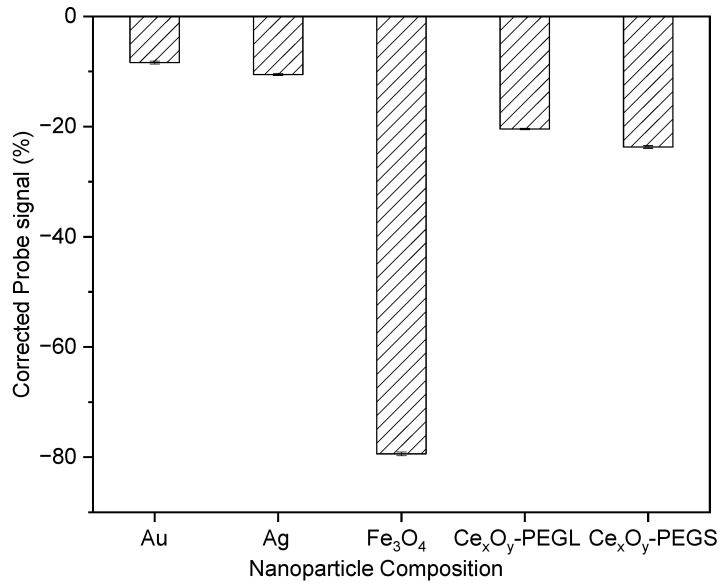
Adjusted TEAC-fluorescein assay outcomes after compensating for nanoparticle-fluorescein interactions. Across all nanoparticle types, the apparent antioxidant capacity decreases after this correction. Notably, iron oxide nanoparticles show pronounced fluorescein quenching, leading to a markedly lower corrected antioxidant capacity.

Sodium fluorescein proved suitable for assessing nanoparticle antioxidant activity, provided that raw fluorescence traces were corrected for nanoparticle-induced quenching [62]. As illustrated in Figure 5A, Trolox produced a distinct induction period, consistent with its strong antioxidant reactivity. Control experiments confirmed that Trolox itself did not alter probe emission in the absence of oxidant. In contrast, ceria nanoparticles caused a concentration-dependent decrease in fluorescein emission even without oxidant addition (Figure 5B), likely due to probe adsorption on the particle surface. To compensate, time-dependent signals were corrected by subtracting this intrinsic quenching (Figure 5C and Supplemental Figure S1). Following correction, nanoparticle suspensions exhibited weaker reactivity than Trolox at comparable concentrations, showing only delayed oxidation rather than a clear induction time. Nevertheless, concentration-dependent analysis (Supplementary Figure S2) revealed a higher overall antioxidant capacity for ceria compared with Trolox (Figure 5D). Without correction for nanoparticle quenching, the antioxidant capacity would be overestimated. The magnitude of this interference differed among particle types and was most pronounced for transition metal nanoparticles, consistent with their known fluorescence-quenching behavior (Figure 6).

## 4. Discussion

**Figure 7:**
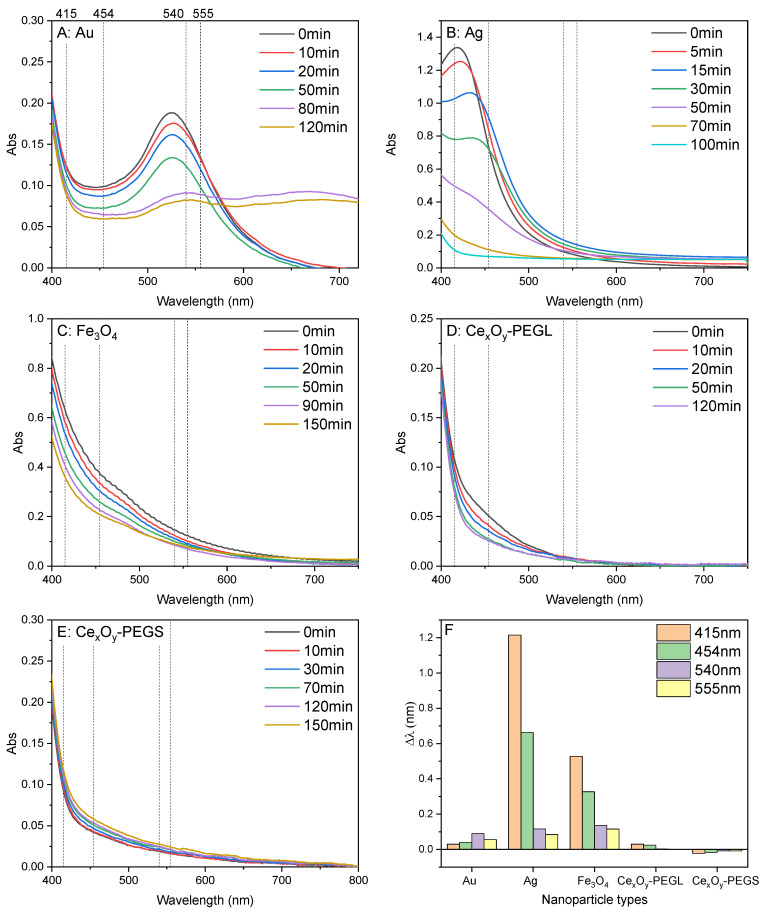
Changes in the absorption spectra of nanoparticles during the oxidation process induced by AAPH at 37 °C: (**A**) Au, (**B**) Ag, (**C**) Fe_3_O_4_, (**D**) Ce_x_Oᵧ-PEG-long, and (**E**) Ce_x_Oᵧ-PEG-short. (**F**) Relative absorbance intensities of the nanoparticles at four wavelengths (415 nm, 454 nm, 540 nm, and 555 nm) before and after AAPH-triggered oxidation at 37 °C.

Spectrophotometric assays are commonly used to evaluate antioxidant activity. However, the intrinsic optical properties of nanoparticles can interfere with these assays by altering absorbance during oxidation [13,63,64]. Similar issues have previously been documented for strongly colored molecules such as tannins. As illustrated in Figure 7, the nanoparticles examined in this work display significant absorption at wavelengths typically selected for probe oxidation measurements, and their spectra further evolve upon reaction with oxidizing agents. While this feature allows direct monitoring of nanoparticle oxidation kinetics by reactive oxygen species and thereby offers insight into their antioxidant behavior, it simultaneously introduces challenges for the use of conventional assays. Standard methods assume that absorbance changes at a set wavelength result only from probe oxidation. However, oxidation of nanoparticles can also alter the outcome. Whether this effect leads to an under- or overestimation of antioxidant capacity relative to Trolox depends on the direction of absorbance change associated with the probe’s oxidation.

**Figure 8:**
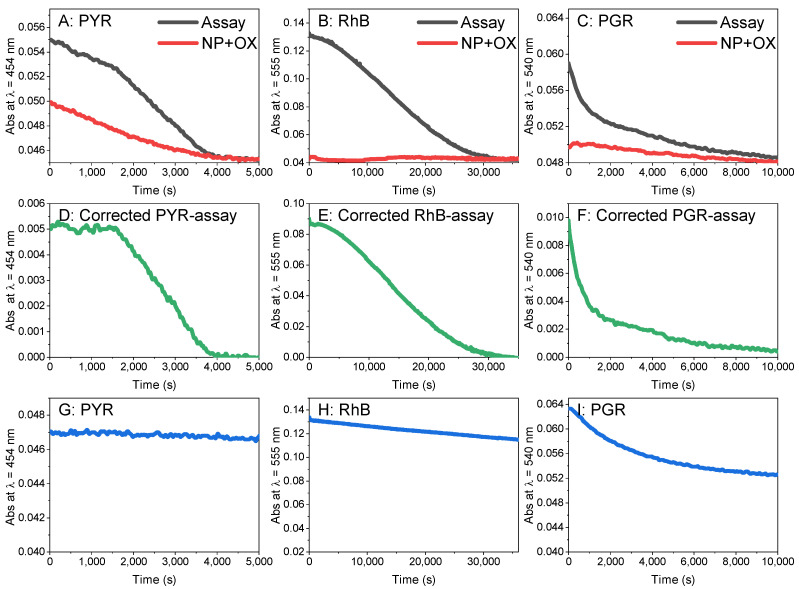
UV-absorbance profiles of different probes during oxidation and control experiments: (**A**) Pyranine (20 µL, 10 µM in well solution), (**B**) pyrogallol red (20 µL, 10 µM in well solution), (**C**) rhodamine B (20 µL, 15 µM in well solution), consumption (Black) triggered by AAPH (10 mM, 80 µL in PYR; 50 mM, 80 µL in RhB; 20 mM, 20 µL in PGR) decomposition at 37 °C under the protection of Ce_x_O_y_-PEG-long (50 ppm) nanoparticles (100 µL in PYR and RhB; 150 µL in PGR); UV-absorbance change in Ce_x_O_y_-PEG-long (50 ppm) nanoparticles at desired wavelength ((**A**): 454 nm, (**B**): 555 nm, (**C**): 540 nm) during the oxidation triggered by AAPH decomposition at 37 °C without the participation of probes. Green lines ((**D**): pyranine, (**E**): pyrogallol red, and (**F**): rhodamine B) suggest corrected probe consumptions after deducting the effect from nanoparticle oxidation. Blue lines show probe ((**G**): pyranine, (**H**): pyrogallol red, and (**I**): rhodamine B) auto-oxidation at 37 °C without the participation of AAPH or nanoparticles. The final solution volume in each well was adjusted to 250 µL using PBS buffer.

**Figure 9:**
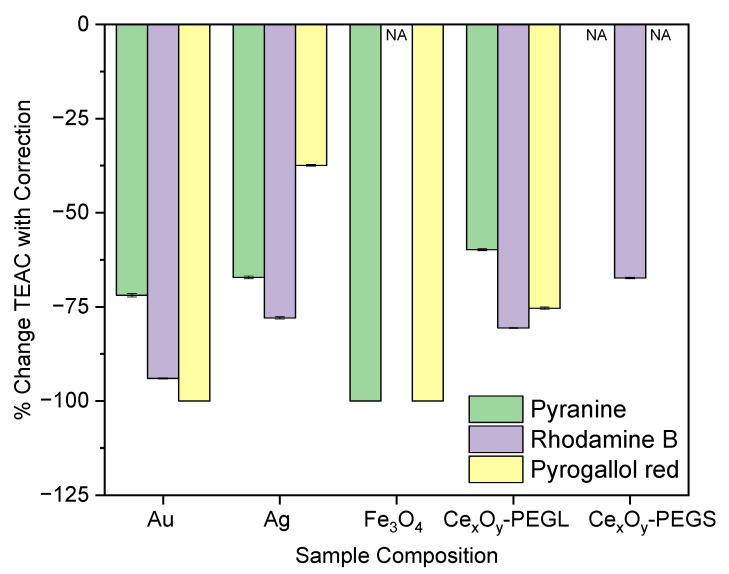
Changes in Trolox-standard assay results after correcting for nanoparticle absorbance variations. These adjustments account for both probe–nanoparticle interactions and absorbance shifts caused by nanoparticle oxidation. For several samples labeled “NA,” corrected measurements could not be obtained due to assay limitations.

Two main approaches can be employed to address the issue of absorbance interference. The first and most direct method involves correcting the probe’s time-resolved optical density at the monitoring wavelength by accounting for the contribution from nanoparticle oxidation alone. This procedure is demonstrated in Figure 8 using ceria nanoparticles as an example. In this case, the red curves represent the absorption changes arising exclusively from the oxidant, AAPH. The black curves correspond to the mixture of oxidant, nanoparticle, and probe, thus incorporating both probe oxidation and nanoparticle oxidation. Subtracting the nanoparticle contribution from the composite signal yields a clearer picture of probe oxidation dynamics. For instance, in Figure 8D, correction of the pyranine data removes the modest absorbance changes caused by nanoparticle oxidation, thereby revealing a distinct induction period that is not apparent in the raw signal (Figure 8A). This adjustment enables a more reliable comparison of nanoparticle-mediated effects on probe oxidation with those produced by a molecular antioxidant standard, such as Trolox. As shown in Figure 9, applying this correction consistently reduces the apparent antioxidant capacity relative to Trolox. The magnitude of this effect depends strongly on both the nanoparticle type and the probe wavelength. Notably, silver nanoparticles present the greatest challenge because their dissolution generates turbid, highly scattering solutions that complicate accurate measurements.

An alternative approach to reduce nanoparticle interference is to monitor probe oxidation at a different wavelength. For instance, in the pyranine assay, nanoparticle oxidation substantially affects optical density at 454 nm (Figure 8A), whereas at 555 nm, the wavelength used for Rhodamine B, the nanoparticle contribution is negligible (Figure 8C). When the probe exhibits sufficient absorbance changes at the selected wavelength, this method allows a reliable relative evaluation of nanoparticle antioxidant activity. The ABTS [2,2′-Azino-bis(3-ethylbenzothiazoline-6-sulfonic acid)] assay exemplifies this strategy, as its absorbance can be monitored well into the red region. Detection wavelengths up to 720 nm have been employed to minimize interference from strongly colored molecular antioxidants, such as tannins.

An additional consideration emerging from the control experiments is the potential for thermal degradation of the probe over time, which can alter optical signals even in the absence of oxidants. This issue is not specific to nanoparticle testing and may affect any assay. Most assays employed AAPH to generate radicals at 37 °C. Under these conditions, pyranine absorption remained unchanged (Figure 8G), whereas rhodamine B exhibited minor variations, and pyrogallol red underwent noticeable decay (Figure 8H,I). Since this thermal effect similarly influences both nanoparticle and Trolox standard measurements, explicit correction is generally unnecessary. However, the rapid decay of pyrogallol red limits its utility for assessing slow-reacting antioxidants, such as ceria nanoparticles, rendering this probe unsuitable for characterizing these materials under the current conditions. Lowering the assay temperature could potentially slow probe auto-oxidation and expand its applicability.

After accounting for various nanoparticle interferences, antioxidant capacities were evaluated using Michaelis–Menten plots of the time-dependent data versus concentration. Temporal oxidation was quantified using the area under the curve (AUC), and linear relationships between AUC and the concentration of Trolox or nanoparticles were established (Supplementary Figures S3–S10) [42,52]. The choice of concentration unit for comparing nanomaterials with molecular antioxidants is nontrivial. In the case of a molecular antioxidant, the per-molecule unit of molarity is suitable as each antioxidant molecule undergoes a single oxidation event. Alternatively, some researchers prefer reporting potency per-weight, which can be more relevant for therapeutic dosing. Comparisons between molecules are generally unaffected by this choice, because their molecular weights are similar. Nanoparticles, however, contain multiple oxidation sites and possess molecular weights orders of magnitude higher than Trolox. This makes per-particle or per-mole comparisons potentially misleading. Selection of the concentration metric can thus significantly influence the interpretation of nanoparticle antioxidant capacity. Therefore, it should be selected to match the research objectives (Supplementary Figure S2). Molar or particle-number units are suitable for comparing nanoparticle types, whereas mass-based metrics facilitate comparison between nanoparticles and molecular antioxidants and align with applications where ppm dosing is relevant. In this study, we adopted a mass-based metric for comparison with Trolox and to enable practical cross-comparisons among nanoparticles.

**Figure 10:**
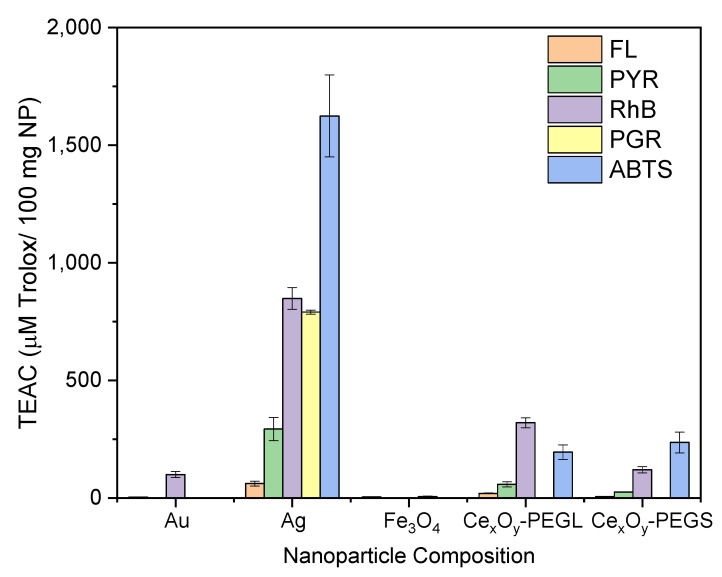
Antioxidant capacities of the five types of nanoparticle types, expressed relative to Trolox, as determined by multiple assay methods.

Figure 10 presents the relative antioxidant capacities of five types of nanoparticle types versus Trolox across the different assays, while Table 3 provides a rank order of their effectiveness. Supplementary Tables S4 and S5 provide detailed summaries. All data were standardized to concentration-dependent oxidation kinetics relative to Trolox (“Trolox Equivalents”). Across assays, rank ordering of particle types was consistent: silver nanoparticles exhibited the highest protective effect on probes, followed by ceria nanoparticles. The superior performance of silver is attributed to oxidative dissolution, providing an effective reactive oxygen species sink, whereas ceria nanoparticles are well-established antioxidants. These findings demonstrate that, when appropriately corrected for interference, conventional antioxidant assays can effectively evaluate and compare the relative potency of nanoparticle-based antioxidants.

These assays are best suited for semi-quantitative evaluations, since their absolute quantification of antioxidant capacity is strongly influenced by the particular probe/oxidant pair employed (Figure 10). For example, depending on the assay, silver nanoparticles can exhibit activities ranging from about 60- to 500-fold higher than Trolox. The most active ceria nanoparticles show enhancements of roughly 20- to 100-fold compared with Trolox, depending on the probe used. Such variability is consistent with prior reports. Comparative analyses of molecular antioxidants across different assays similarly reveal large discrepancies in quantitative outcomes, which can be attributed to the distinct reactivity of probes toward oxidants [65]. Thus, although conventional antioxidant assays may not provide strictly quantitative comparisons when used side by side as in this work, they nevertheless supply compelling evidence for the strong antioxidant activity of nanoparticles under diverse conditions. Moreover, they facilitate cross-comparison among nanoparticle types, offering guidance for tailoring nanoparticle structures to maximize antioxidant performance.

## 5. Conclusions

This study demonstrates that conventional spectrophotometric assays can be adapted to evaluate nanoparticle antioxidant activity despite optical interferences from absorption, scattering, and dissolution. By applying signal correction and wavelength selection strategies, we achieved more reliable comparisons with the molecular standard Trolox. Across assays, silver and ceria nanoparticles consistently exhibited the strongest antioxidant capacities, confirming their high reactivity toward reactive oxygen species. These results emphasize the promise of nanoparticles as effective antioxidants and offer practical insights for enhancing assay reliability and comparability in future research.

## Figures and Tables

**Table 1:** Traditional antioxidant assays for determining nanoparticle antioxidant capacity. The 2,2-diphenyl-1-(2,4,6-trinitrophenyl)-hydrazinyl (DPPH) assay, 2,2′-azino-bis(3-ethylbenzothiazoline-6-sulfonic acid) (ABTS) assay, and sodium fluorescein (NaFluo) assay are among the most widely used methods for measuring Trolox equivalent antioxidant capacity (TEAC) assays. However, each technique exhibits certain limitations when applied to nanoparticle systems. Here, “prevalence” denotes the number of publications retrieved from Web of Science that list both the probe and “antioxidant” as indexed topics. Methods categorized as “absorbance ±” rely on changes in absorbance, whereas the fluorescein assay tracks fluorescence decay.

Probe	Oxidant	Method	Example	Prevalence
Pyranine	AAPH	Absorbance−	Gregorio 2020 [29]	27
Pyrogallol red	AAPH	Absorbance−	Bhat 2001 [28]	110
Rhodamine B	AAPH	Absorbance−	Yu 2008 [33]	140
Fluorescein	AAPH	Fluorescence−	Roy 2010 [32]	722
DPPH	NA	Absorbance−	Mensor 2001 [30]	33,004
ABTS	Fe^2+^/H_2_O_2_	Absorbance+	Pellegrini 1999 [31]	11,331

**Table 2:** Analytical results for the TEAC-PYR microplate method using different AAPH and probe concentrations for two types of ceria nanoparticles. Pyranine oxidation rates and radical generation rates change with different concentrations of oxidants and pyranine: higher concentrations of oxidants require less reaction time but generate modestly less precision in the linear response. The rate of radical production from AAPH thermolysis, or *R_i_*, was calculated from the induction time (e.g., *t*_ind_ (s) = *n* [HX]/*R*_i_, where [HX] is the antioxidant concentration, and *n* represents the number of radicals neutralized by each antioxidant molecule. The value of n for Ce_x_O_y_-DOPA-PEG 10 K (CexOy-10 K) and Ce_x_O_y_-DOPA-PEG 5 K (Ce_x_O_y_-5 K) was calculated assuming an *n* = 2 for Trolox. The factor *n* increases when probe concentration increases, while it decreases when radical concentration increases. The shorter molecular weight polymer coatings resulted in slightly more radical quenching capacity per particle.

AAPH (mM)	Linear Range (μM)	PYR (μM)	*R*_i_ (μM/min)	Radicals Quenched by Ce_x_O_y_-10 K	Radicals Quenched by Ce_x_O_y_-5 K	Analysis Time (min)	Intercept (min)	*R* ^2^
5	0–20	5	0.109	--	97.1	340	8.7 ± 9.0	0.998
		10	0.128	40.0	53.2	340	6.1 ± 13.6	0.997
		20	0.122	43.0	--	440	4.7 ± 10.5	0.997
10	0–20	5	0.188	--	82.0	200	5.1 ± 4.6	0.998
		10	0.219	36.8	--	420	9.5 ± 7.2	0.999
		20	0.228	39.0	--	180	6.4 ± 5.3	0.999
		25	0.188	--	--	200	6.6 ± 6.9	0.996
15	0–20	15	0.262	--	66.4	150	3.3 ± 3.4	0.998
20	0–20	10	0.380	30.5	--	420	1.7 ± 3.8	0.997
		20	0.424	33.2	--	230	2.4 ± 1.8	1.000
50	0–100	10	0.913	25.4	--	110	0.6 ± 0.6	1.000
		20	1.040	25.8	--	100	1.3 ± 1.1	0.999
100	0–100	25	1.965	--	--	100	2.9 ± 3.4	0.993

**Table 3:** Rank order of antioxidant capacities after correction for optical interferences. Data are reported as the mass of nanoparticles relative to the mass of Trolox equivalents. A dash indicates unavailable results.

Nanoparticles	TEAC-ABTS	TEAC-PYR	TEAC-RhB	TEAC-PGR	TEAC-FL
Au	--	--	3	--	5
Ag	1	1	1	1	1
Fe_3_O_4_	--	--	--	--	4
Ce_x_O_y_-PEGL	2	2	2	2	2
Ce_x_O_y_-PEGS	3	3	4	--	3

## Data Availability

Data will be made available on request.

## Supplementary Materials

The following supporting information can be downloaded at: https://www.mdpi.com/article/10.3390/antiox14101254/s1, Figure S1: Histogram of nanoparticles used in the antioxidant capacity measurement; Figure S2: TEAC of five types of nanoparticles before and after correction; Figure S3: Sodium fluorescein consumption caused by interaction with nanoparticles and oxidation triggered by AAPH decomposition under 37 ℃ under the protection of nanoparticles; Figure S4: Corrected sodium fluorescein consumption caused by interaction with nanoparticles and oxidation triggered by AAPH decomposition under 37 ℃ under the protection of nanoparticles; Figure S5: Nanoparticle absorbance change caused by oxidation and pyranine consumption triggered by AAPH decomposition under 37 ℃ under the protection of nanoparticles; Figure S6: Corrected pyranine consumption caused by interaction with nanoparticles and oxidation triggered by AAPH decomposition under 37 ℃ under the protection of nanoparticles; Figure S7: Nanoparticle absorbance change caused by oxidation and rhodamine B consumption triggered by AAPH decomposition under 37 ℃ under the protection of nanoparticles; Figure S8: Corrected rhodamine consumption caused by interaction with nanoparticles and oxidation triggered by AAPH decomposition under 37 ℃ under the protection of nanoparticles; Figure S9: Nanoparticle absorbance change caused by oxidation and pyrogallol red consumption triggered by AAPH decomposition under 37 ℃ under the protection of nanoparticles; Figure S10: Corrected pyrogallol red consumption caused by interaction with nanoparticles and oxidation triggered by AAPH decomposition under 37 ℃ under the protection of nanoparticles; Figure S11: Pyranine consumption caused by radical oxidation triggered by AAPH under 37 ℃ under the protection of Trolox; Figure S12: Pyranine consumption caused by radical oxidation triggered by AAPH under 37 ℃ under the protection of Trolox; Figure S13: Calibration of the induction time (Tind) of pyranine consumption caused by AAPH thermolysis under 37 ℃ under the protection of Trolox at different pyranine and AAPH concentration; Figure S14: Calibration of the induction time (Tind) of pyranine consumption caused by AAPH thermolysis under 37 ℃ under the protection of Trolox at different pyranine and AAPH concentration; Figure S15: Pyranine consumption caused by radical oxidation triggered by AAPH at 37 ℃ under the protection of CexOy-PEG-long Experiments measured at different pyranine and AAPH concentrations separately; Figure S16: Pyranine consumption caused by radical oxidation triggered by AAPH at 37 ℃ under the protection of CexOy-PEG-long; Figure S17: Absorbance of ABTS·+ at 415 nm measured 10 mins after the injection of H2O2/myoglobin solution under the protection of five types of nanoparticles; Table S1: Physical properties (diameters, densities, and concentrations) of nanoparticles used in TEAC measurements; Table S2: TEAC of five types of nanoparticles calculated by the area under the curve (AUC) in ORAC assays using fluorescein, pyranine, pyrogallol red, and rhodamine B as probes; Table S3: (Continued) TEAC of five types of nanoparticles calculated by the area under the curve (AUC) in ORAC assays using fluorescein, pyranine, pyrogallol red, and rhodamine B as probes; Table S4: TEAC of five types of nanoparticles calculated by the half-life time (t1/2) in ORAC assays using fluorescein, pyranine, pyrogallol red, and rhodamine B as probes; Table S5: (Continued) TEAC of five types of nanoparticles calculated by the half-life time (t1/2) in ORAC assays using fluorescein, pyranine, pyrogallol red, and rhodamine B as probes.

## Abbreviations

The following abbreviations are used in this manuscript:

DPPH	2,2-diphenyl-1-picrylhydrazyl
AAPH	2,2′-azobis(2-methylpropionamidine) dihydrochloride
ABTS	2,2′-azino-bis(3-ethylbenzothiazoline-6-sulfonic acid)
TEAC	Trolox equivalent antioxidant capacity
ROS	Reactive oxygen species
PGR	Pyrogallol red
PYR	Pyranine
RhB	Rhodamine B
NaFluo	Fluorescein sodium salt
