# Supplementary material for: Quantifying the Antioxidant Capacity of Inorganic Nanoparticles: Challenges and Analytical Solutions
Source: Antioxidants (Basel). 2025 Oct 18;14(10):1254. doi: 10.3390/antiox14101254 (PMC12561802; doi:10.3390/antiox14101254)
Supplement: Supplementary file 1 [file antioxidants-14-01254-s001.zip › antioxidants-3879514-supplementary.pdf]

Supporting Information

Quantifying the Antioxidant Capacity of Inorganic Nanoparticles: Challenges and Analytical Solutions

Yue Hu [1], Qingbo Zhang [1], Zhen Xiao [1], Xiaoting Guo [1], Vivian Ling [1], Yidan Bi [1] and Vicki L. Colvin [1,2,*]

[1] Department of Chemistry, Brown University, Providence, RI 02906, USA; yue_hu@alumni.brown.edu (Y.H.); nanomaterialszh@gmail.com (Q.Z.); zhen_xiao@alumni.brown.edu (Z.X.); xiaoting_guo@alumni.brown.edu (X.G.); vivian_ling@alumni.brown.edu (V.L.); yidan_bi@alumni.brown.edu (Y.B.)
[2] School of Engineering, Brown University, Providence, RI 02906, USA
* Correspondence: vicki_colvin@brown.edu

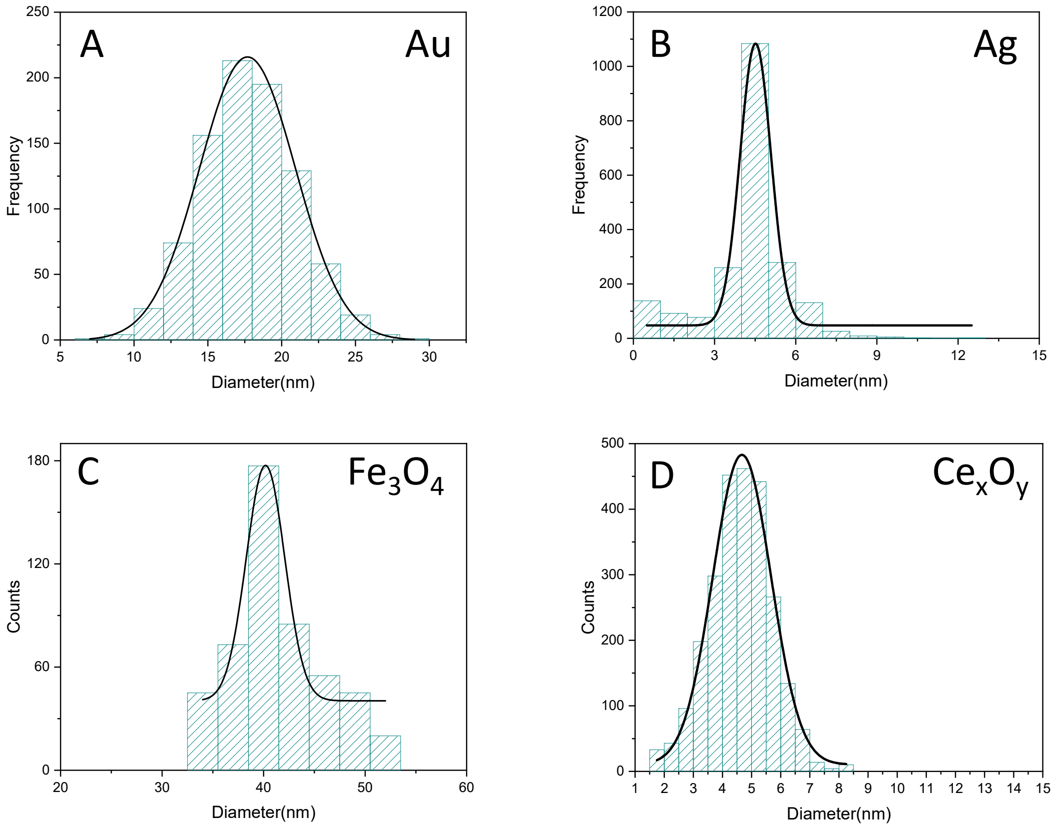

Figure S1. Histogram of nanoparticles used in the antioxidant capacity measurement: A: Gold nanoparticles of 17.7 ± 3.3nm. B: Silver nanoparticles of 4.5 ± 0.6nm. C: Iron oxide nanoparticles with cluster diameter of 40.0 ± 3.0nm and primary particle size of 4.0 ± 0.5nm. D: Ce_xO_y nanoparticles with a diameter of 4.7 ± 1.0nm.

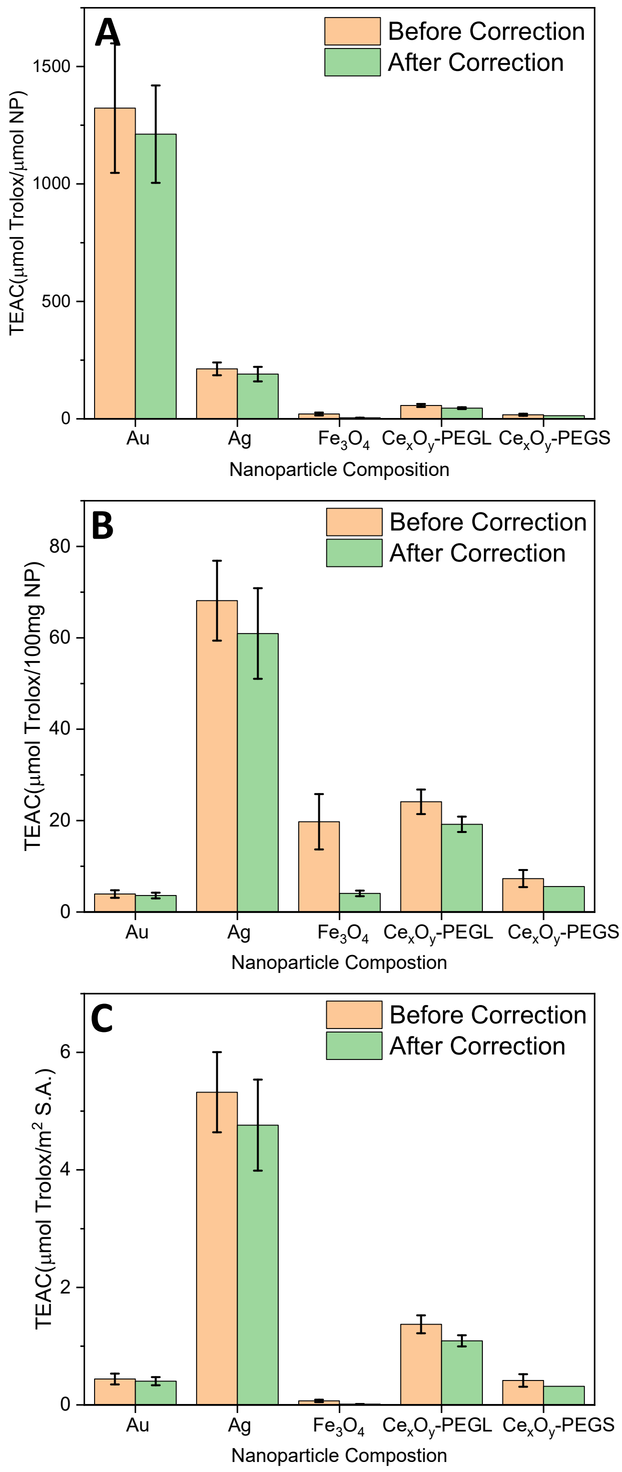

Figure S2. TEAC of five types of nanoparticles before and after correction. Calculated by using the area under the curve (AUC) in sodium fluorescein-ORAC assays presented by (A) per micromole nanoparticle (B) per 100mg nanoparticle, and (C) per 1m^2 of nanoparticle surface area.

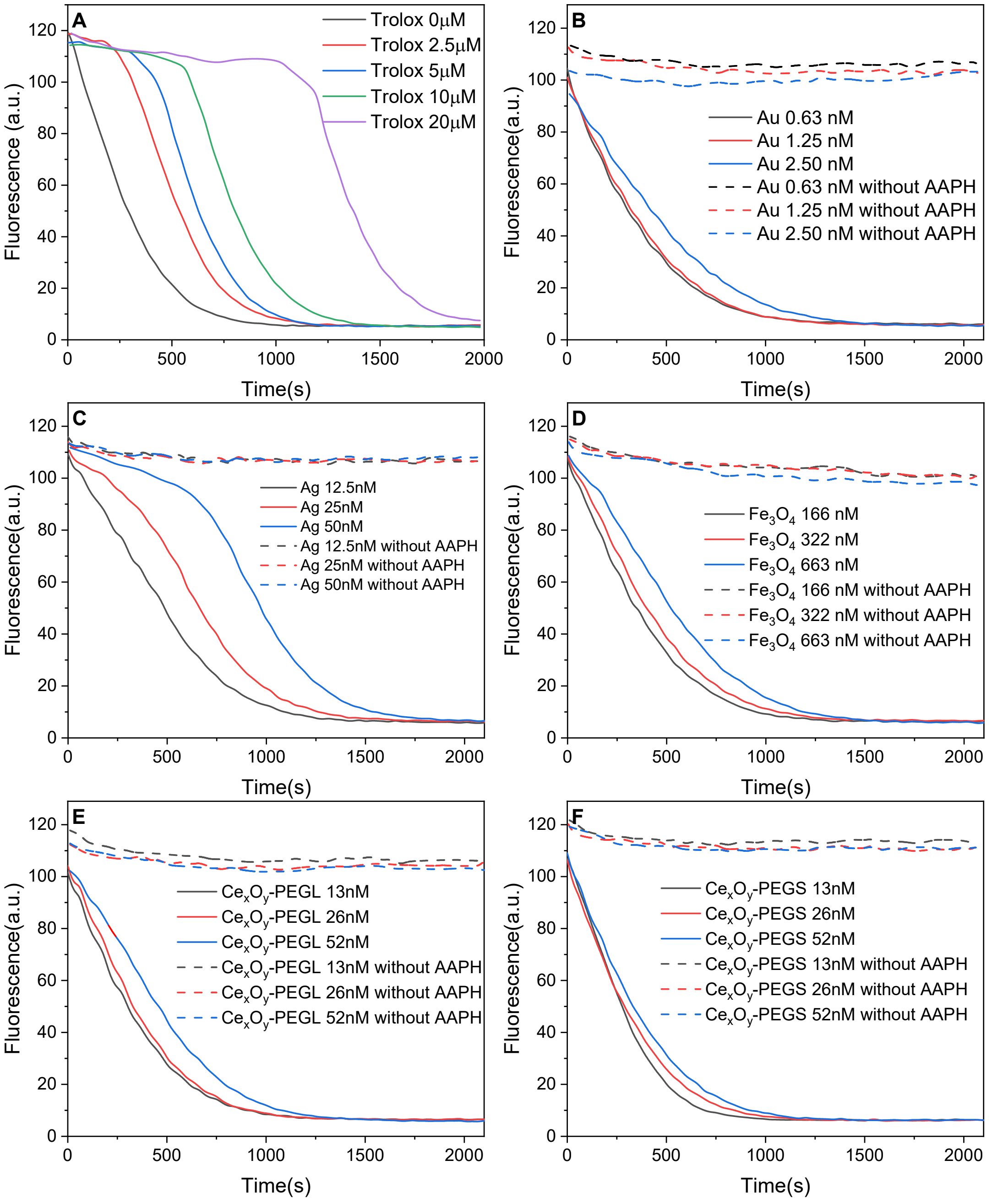

Figure S3. Sodium fluorescein consumption caused by interaction with nanoparticles and oxidation triggered by AAPH decomposition under 37 °C under the protection of nanoparticles: (A) Trolox, (B) gold nanoparticles, (C) silver nanoparticles, (D) iron oxide nanoparticles, (E) Ce_xO_y-PEG-long nanoparticles, and (F) Ce_xO_y-PEG-short nanoparticles.

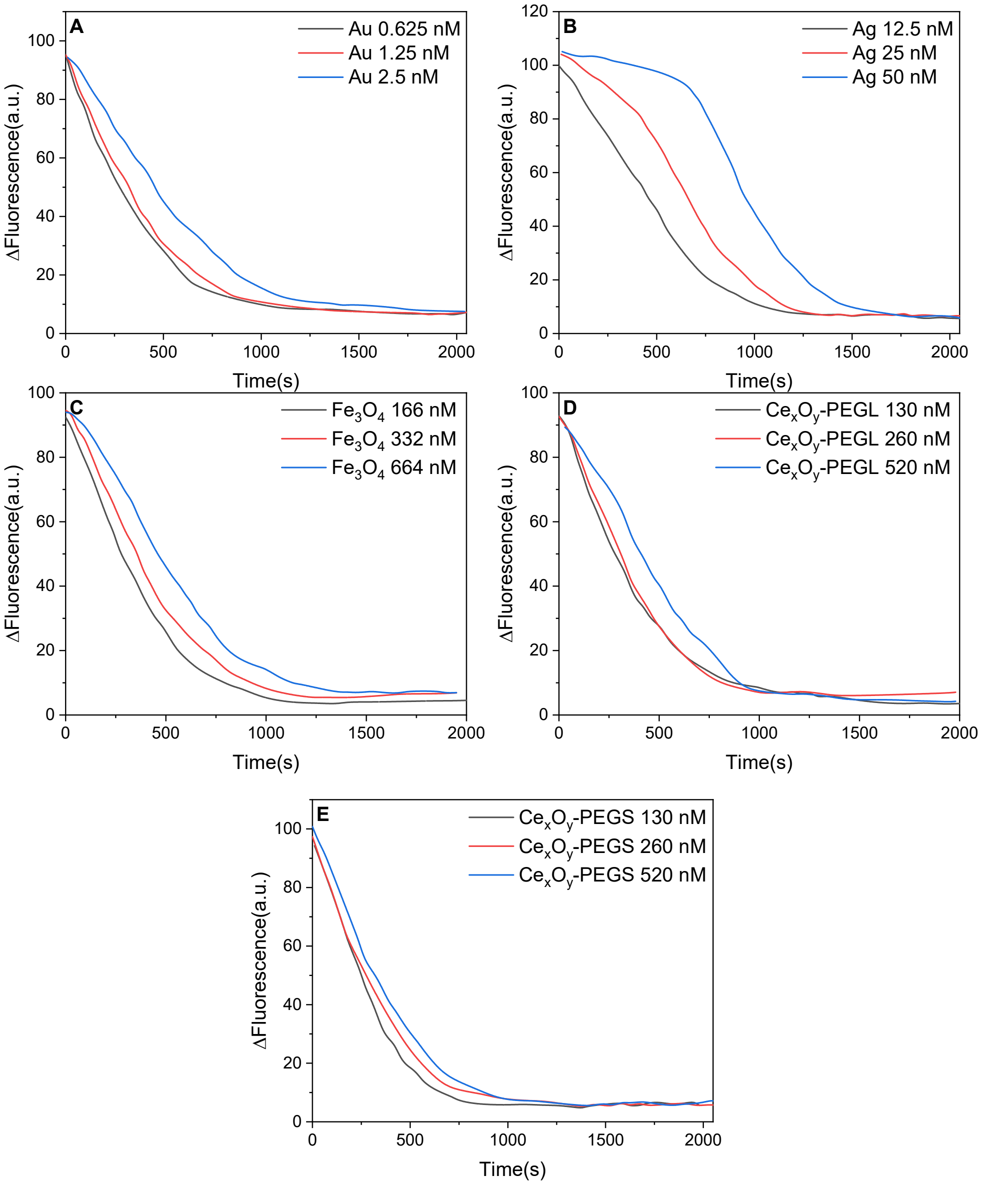

Figure S4. Corrected sodium fluorescein consumption caused by interaction with nanoparticles and oxidation triggered by AAPH decomposition under 37 °C under the protection of nanoparticles: (A) gold nanoparticles, (B) silver nanoparticles, (C) iron oxide nanoparticles, (D) Ce_xO_y-PEG-long nanoparticles, and (E) Ce_xO_y-PEG-short nanoparticles.

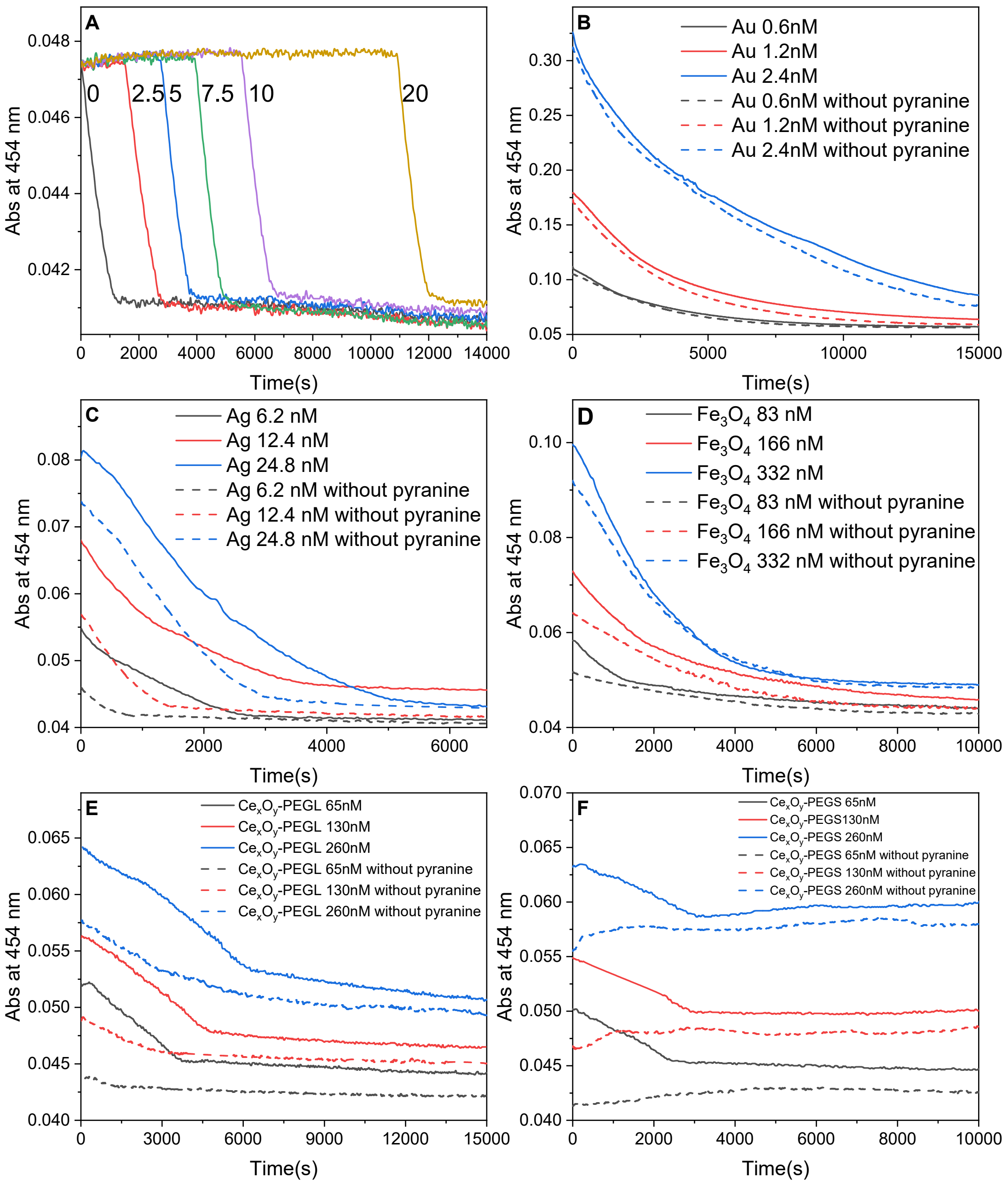

Figure S5. Nanoparticle absorbance change caused by oxidation and pyranine consumption triggered by AAPH decomposition under 37 °C under the protection of nanoparticles: (A) Trolox, (B) gold nanoparticles, (C) silver nanoparticles, (D) iron oxide nanoparticles, (E) Ce_xO_y-PEG-long nanoparticles, and (F) Ce_xO_y-PEG-short nanoparticles. Numbers in panel (A) show the concentrations of Trolox added in µM.

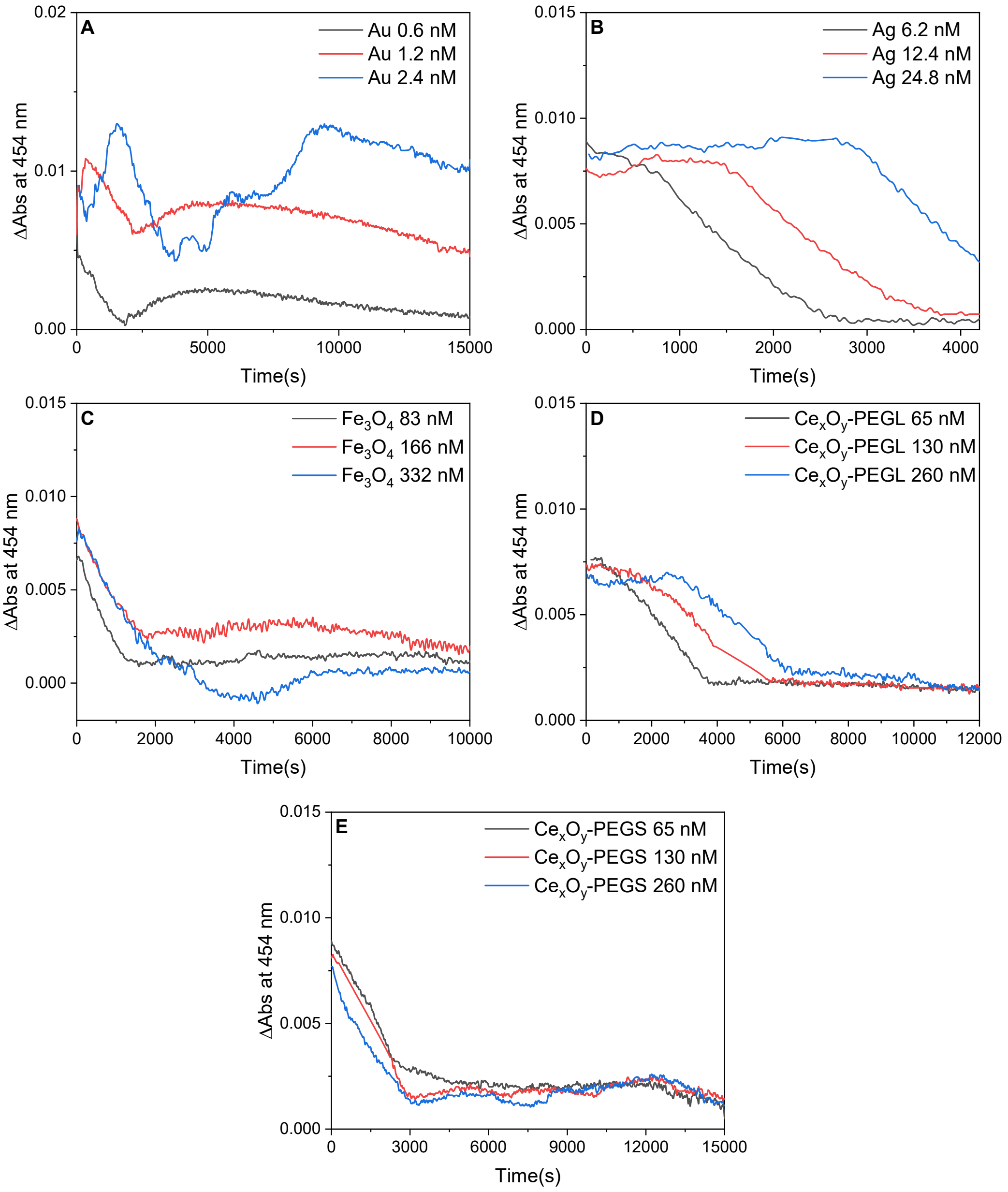

Figure S6. Corrected pyranine consumption caused by interaction with nanoparticles and oxidation triggered by AAPH decomposition under 37 °C under the protection of nanoparticles: (A) gold nanoparticles, (B) silver nanoparticles, (C) iron oxide nanoparticles, (D) Ce_xO_y-PEG-long nanoparticles, and (E) Ce_xO_y-PEG-short nanoparticles. Numbers in panels show the concentrations of nanoparticles added in μM.

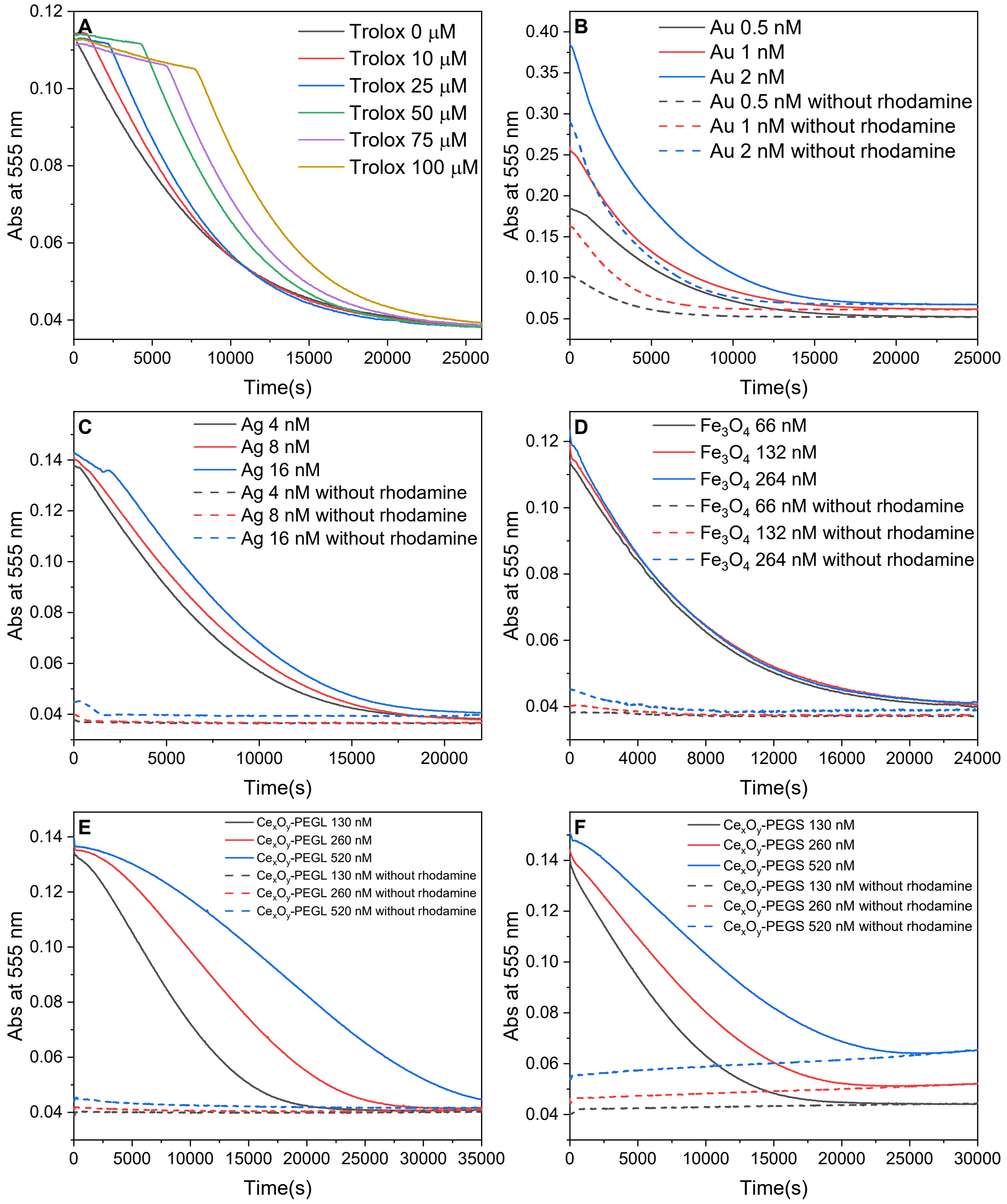

Figure S7. Nanoparticle absorbance change caused by oxidation and rhodamine B consumption triggered by AAPH decomposition under 37 °C under the protection of nanoparticles: (A) Trolox, (B) gold nanoparticles, (C) silver nanoparticles, (D) iron oxide nanoparticles, (E) Ce_xO_y-PEG-long nanoparticles, and (F) Ce_xO_y-PEG-short nanoparticles.

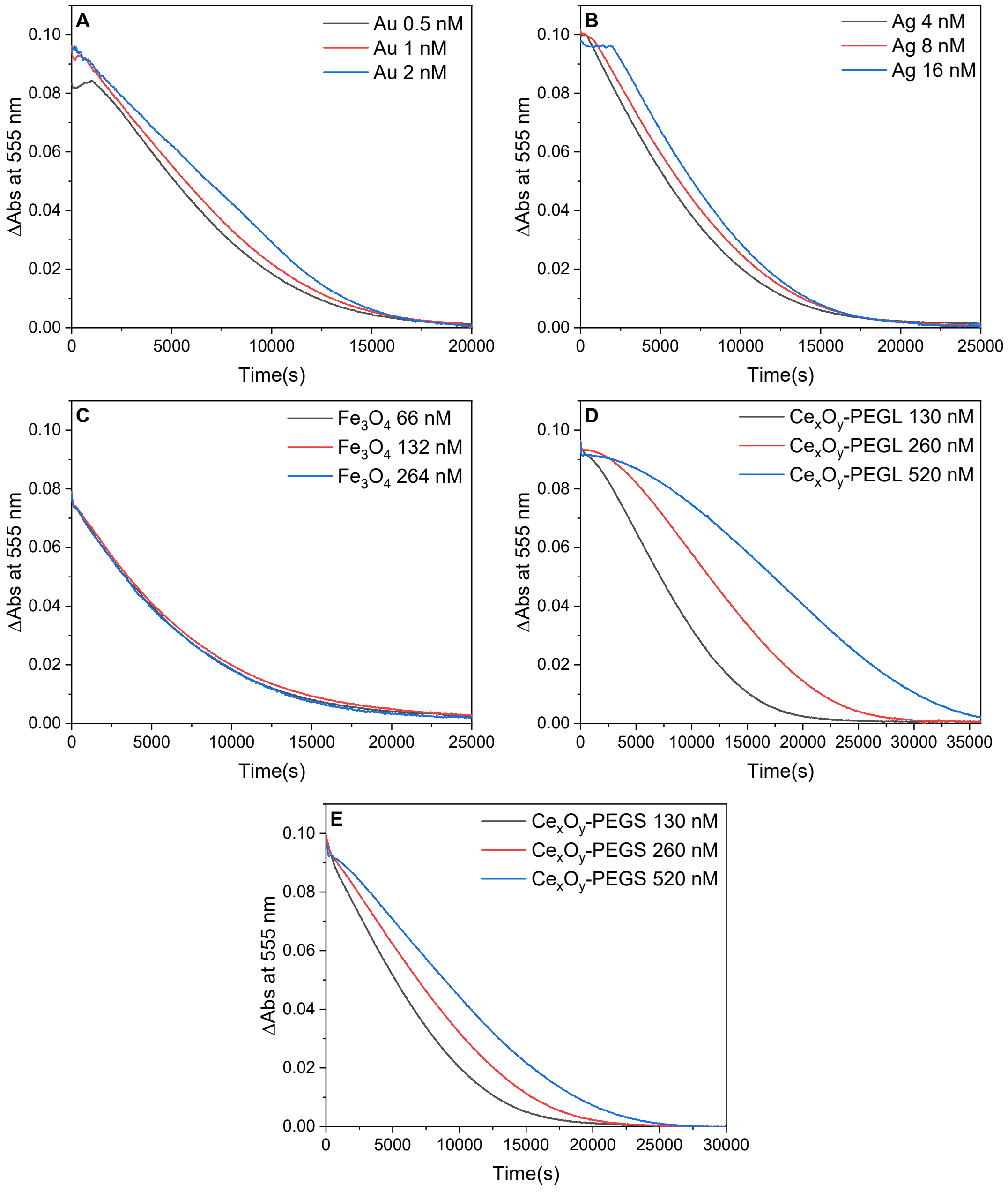

Figure S8. Corrected rhodamine consumption caused by interaction with nanoparticles and oxidation triggered by AAPH decomposition under 37 °C under the protection of nanoparticles: (A) gold nanoparticles, (B) silver nanoparticles, (C) iron oxide nanoparticles, (D) Ce_xO_y-PEG-long nanoparticles, and (E) Ce_xO_y-PEG-short nanoparticles.

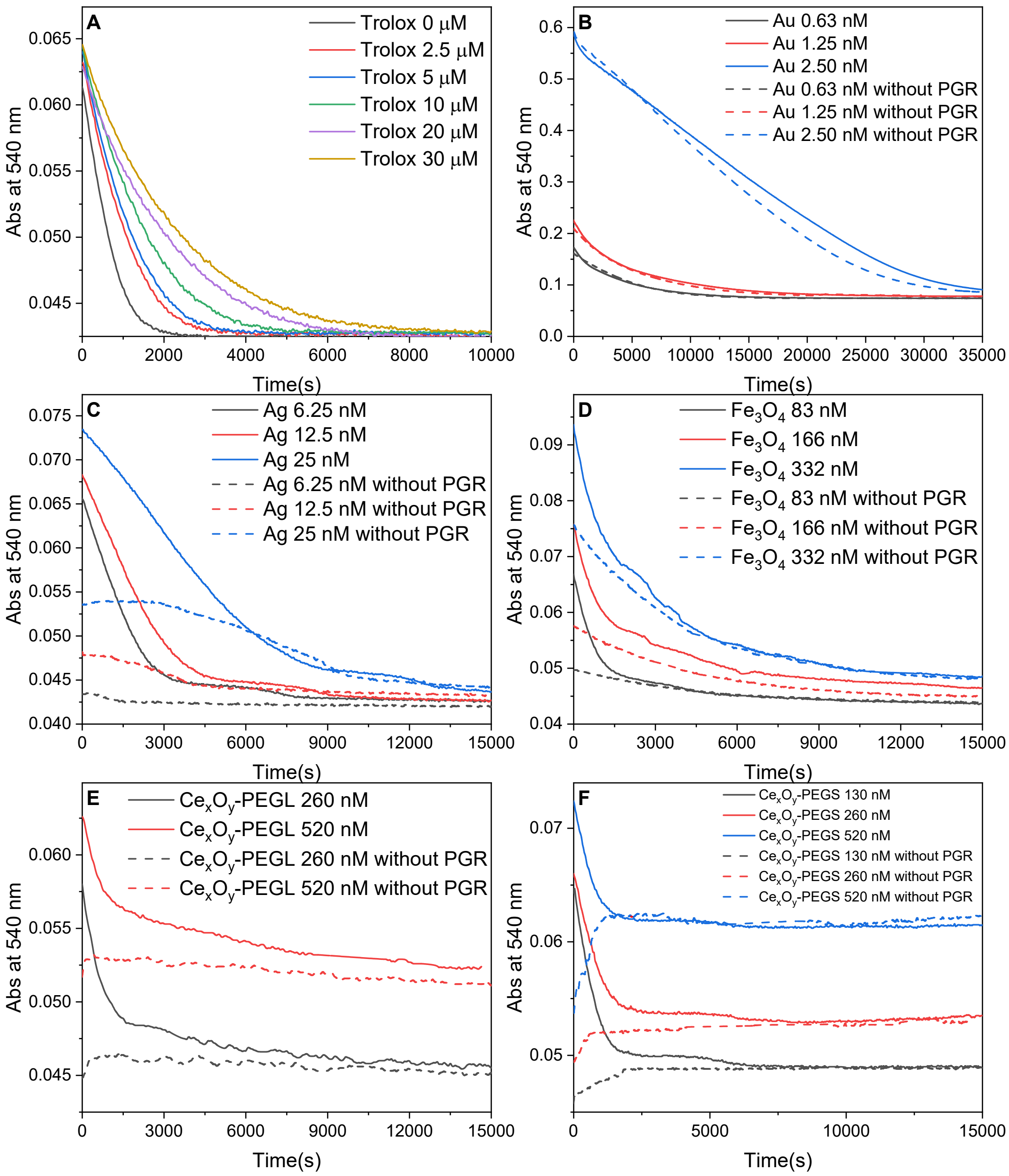

Figure S9. Nanoparticle absorbance change caused by oxidation and pyrogallol red consumption triggered by AAPH decomposition under 37 °C under the protection of nanoparticles: (A) Trolox, (B) gold nanoparticles, (C) silver nanoparticles, (D) iron oxide nanoparticles, (E) Ce_xO_y-PEG-long nanoparticles, and (F) Ce_xO_y-PEG-short nanoparticles.

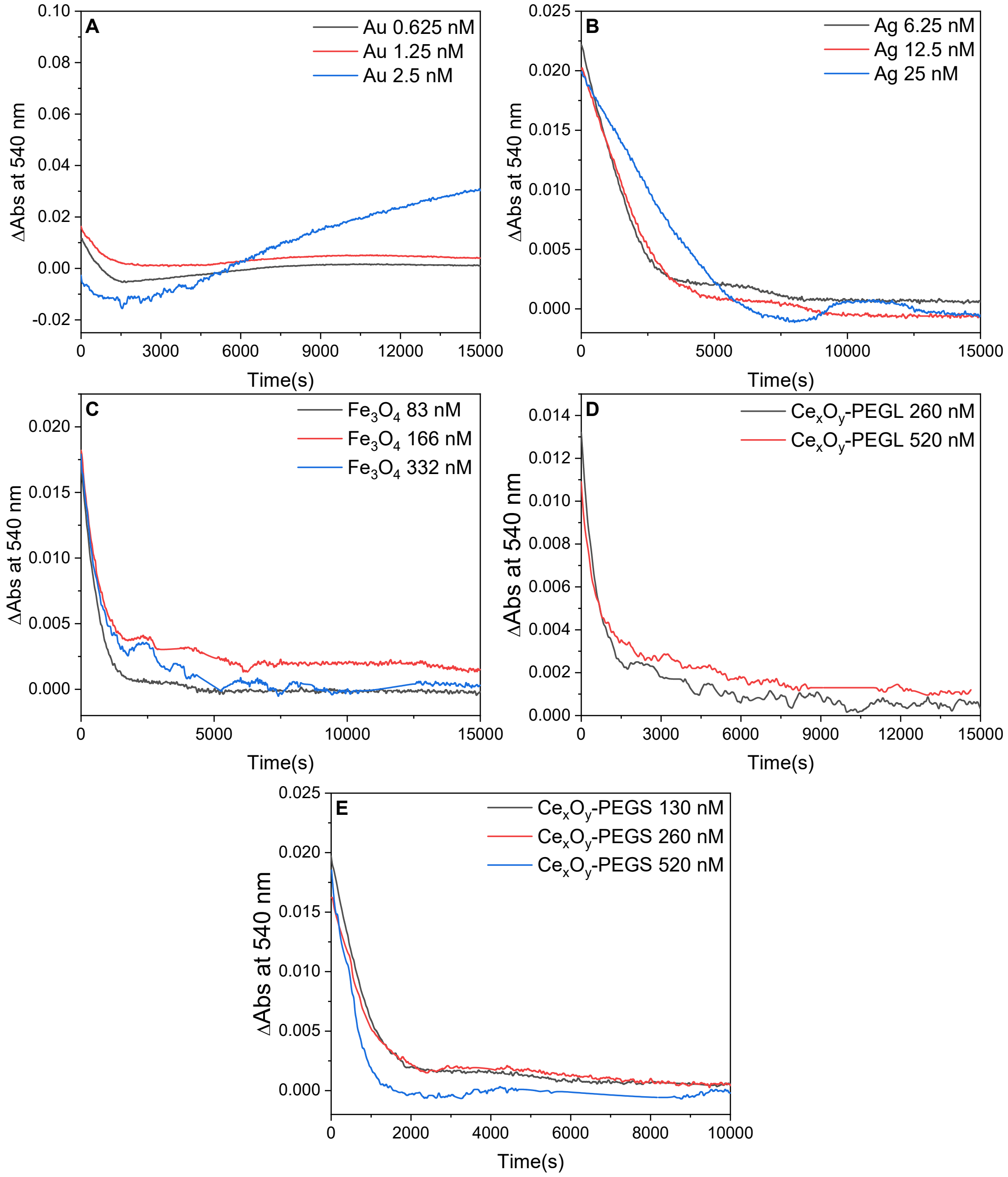

Figure S10. Corrected pyrogallol red consumption caused by interaction with nanoparticles and oxidation triggered by AAPH decomposition under 37 °C under the protection of nanoparticles: (A) gold nanoparticles, (B) silver nanoparticles, (C) iron oxide nanoparticles, (D) Ce_xO_y-PEG-long nanoparticles, and (E) Ce_xO_y-PEG-short nanoparticles.

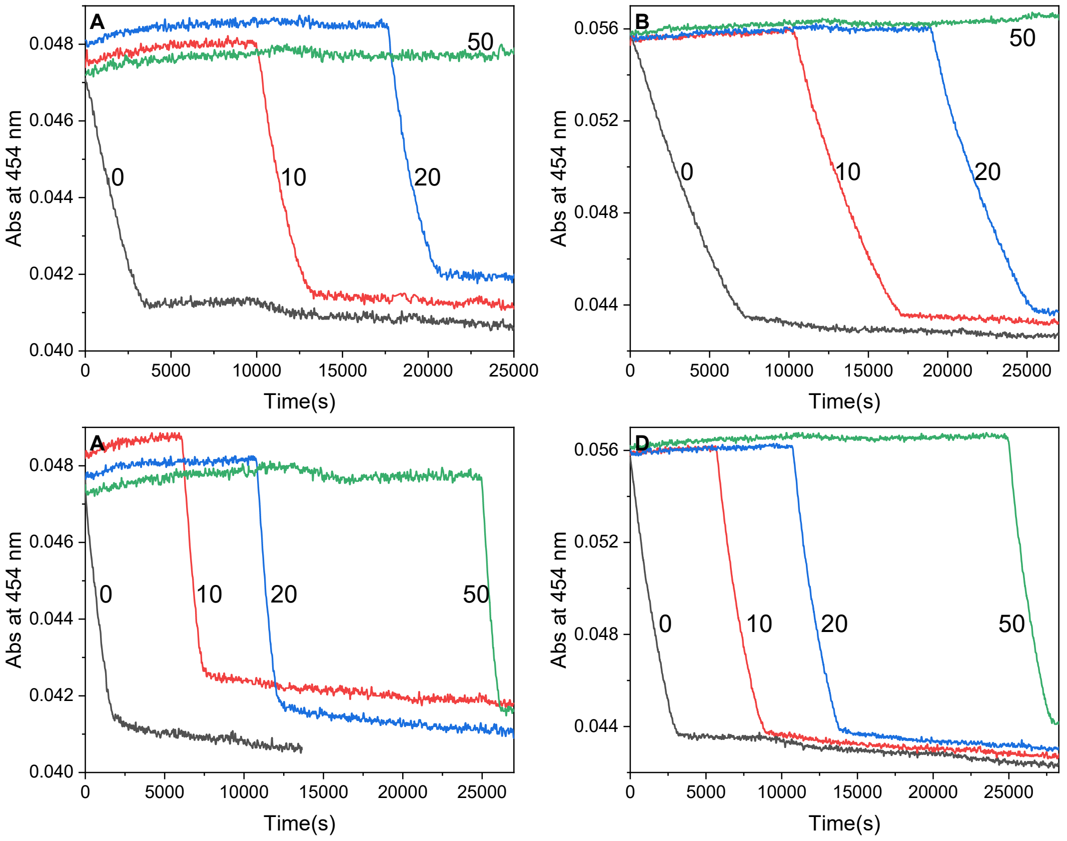

Figure S11. Pyranine consumption caused by radical oxidation triggered by AAPH under 37 ℃ under the protection of Trolox. Pyranine and AAPH were tested at different concentrations: (A) AAPH 5mM, Pyranine 10μM; (B) AAPH 5mM, Pyranine 20μM; (C) AAPH 10mM, Pyranine 10μM; (D) AAPH 10mM, Pyranine 20μM. Numbers in panels show the concentrations of Trolox added in μM.

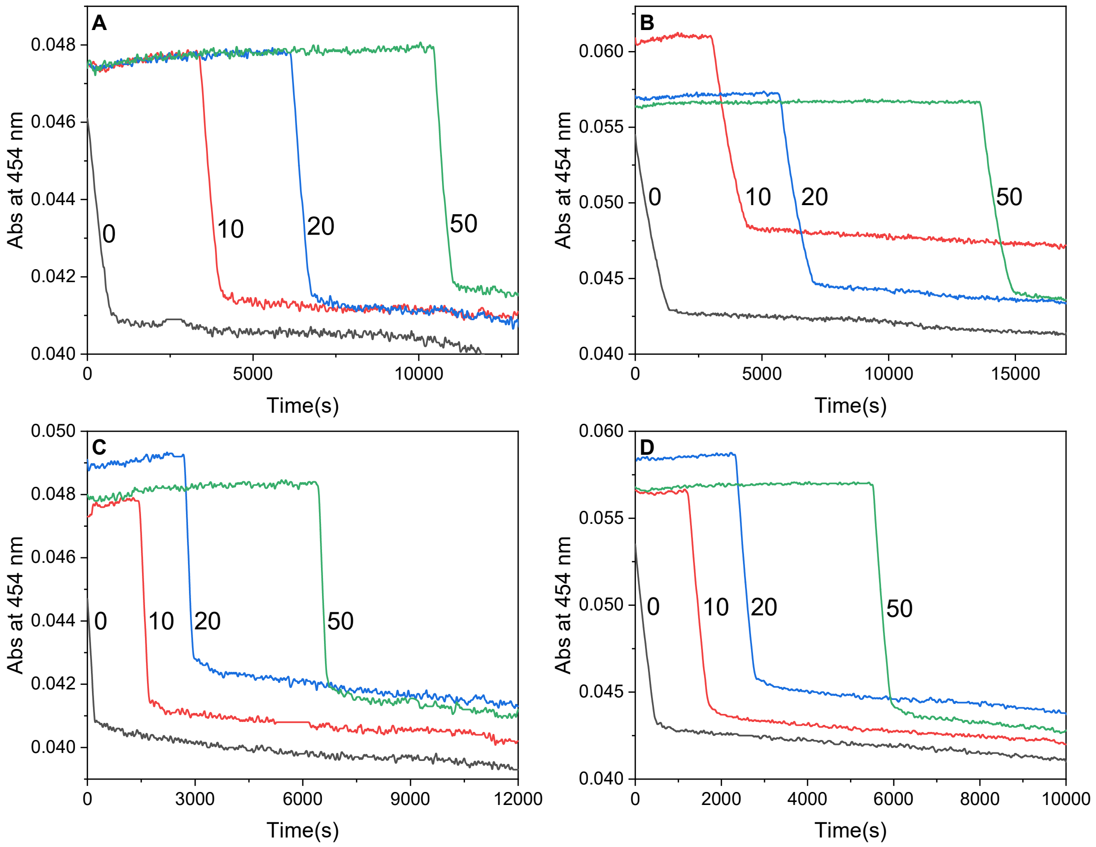

Figure S12. Pyranine consumption caused by radical oxidation triggered by AAPH under 37 ℃ under the protection of Trolox. Pyranine and AAPH were tested at different concentrations: (A) AAPH 20mM, Pyranine 10μM; (B) AAPH 20mM, Pyranine 20μM; (C) AAPH 50mM, Pyranine 10μM; (D) AAPH 50mM, Pyranine 20μM. Numbers in panels show the concentrations of Trolox added in μM.

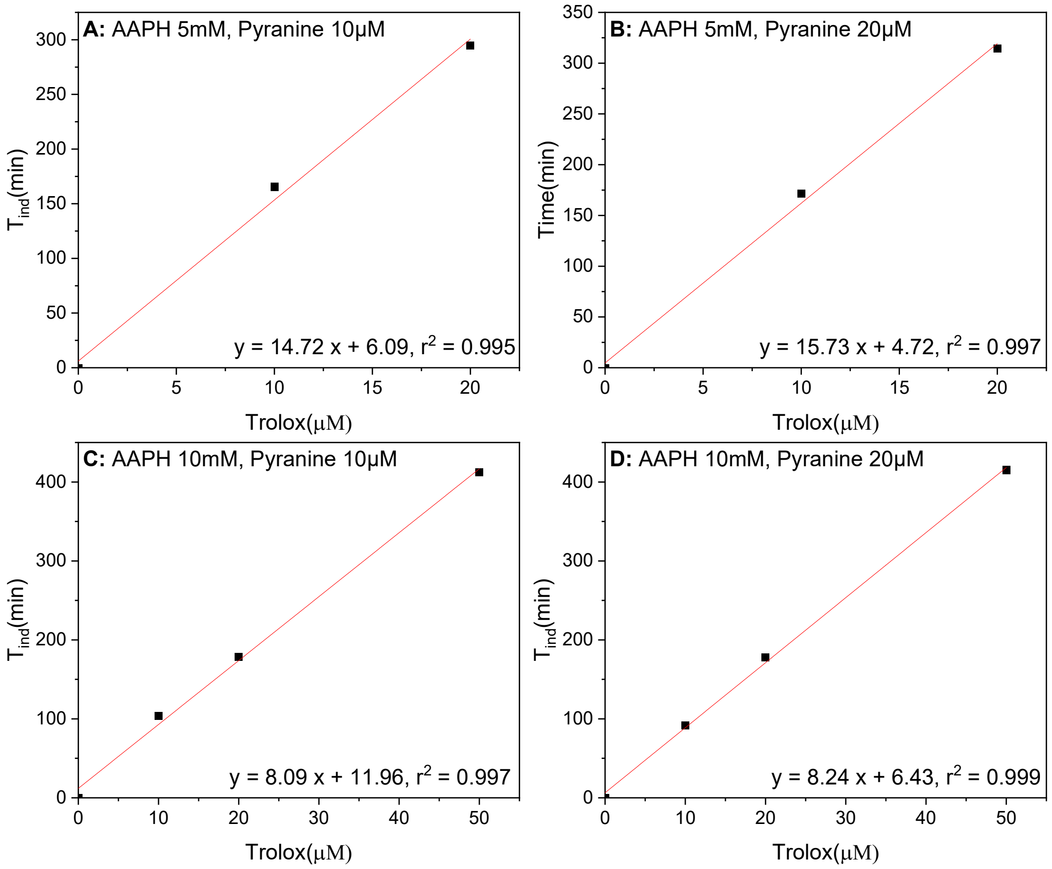

Figure S13. Calibration of the induction time (Tind) of pyranine consumption caused by AAPH thermolysis under 37 °C under the protection of Trolox at different pyranine and AAPH concentration: (A) AAPH 5mM, Pyranine 10μM; (B) AAPH 5mM, Pyranine 20μM; (C) AAPH 10mM, Pyranine 10μM; (D) AAPH 10mM, Pyranine 20μM.

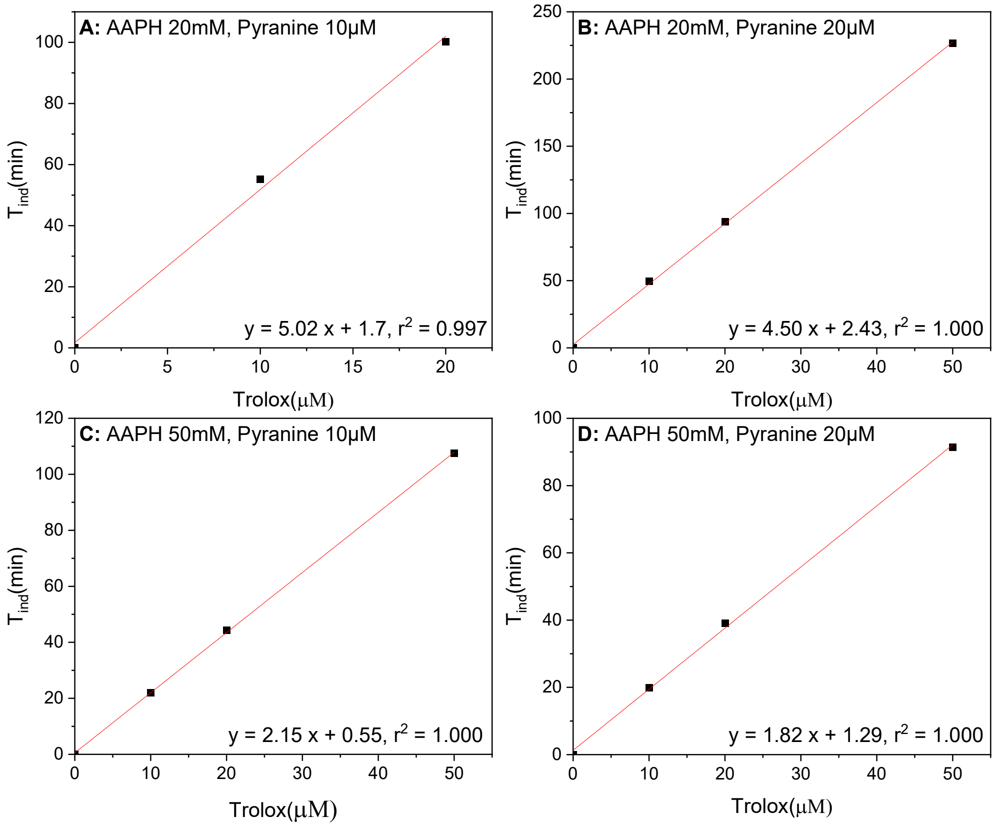

Figure S14. Calibration of the induction time (Tind) of pyranine consumption caused by AAPH thermolysis under 37 °C under the protection of Trolox at different pyranine and AAPH concentration: (A) AAPH 20mM, Pyranine 10μM; (B) AAPH 20mM, Pyranine 20μM; (C) AAPH 50mM, Pyranine 10μM; (D) AAPH 50mM, Pyranine 20μM.

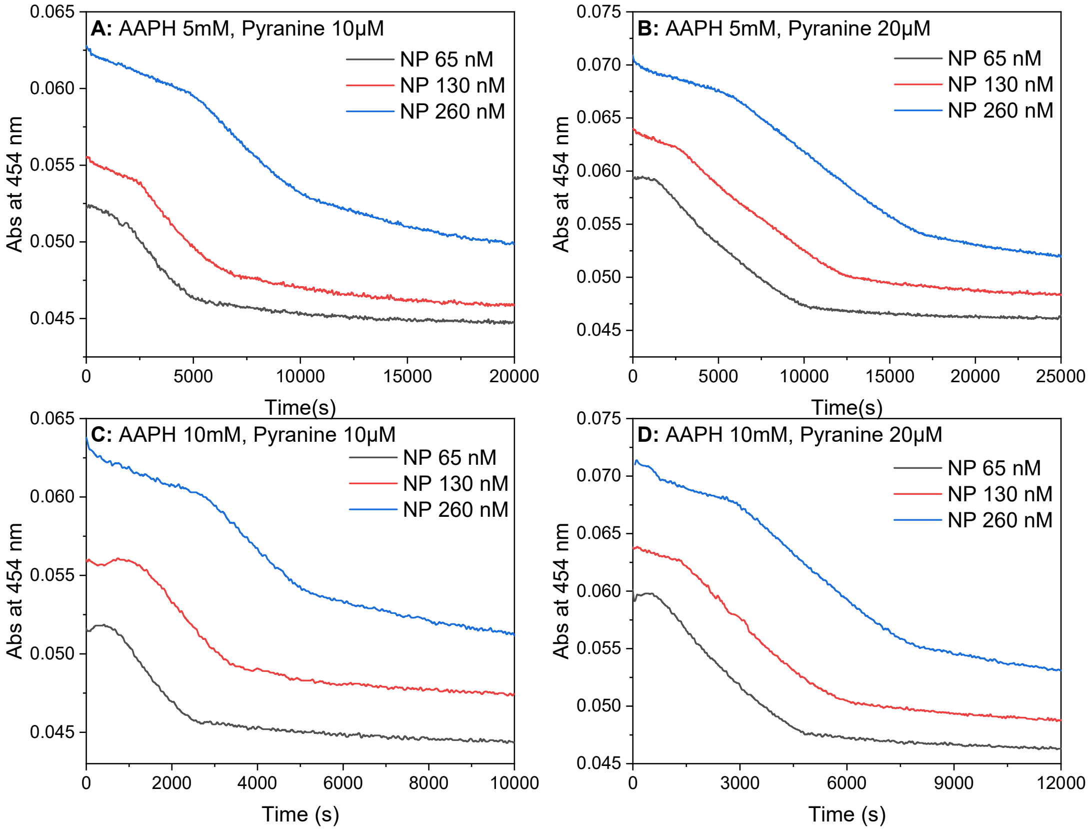

Figure S15. Pyranine consumption caused by radical oxidation triggered by AAPH at 37 °C under the protection of Ce_xO_y-PEG-long. Experiments measured at different pyranine and AAPH concentrations separately: (A) AAPH 5mM, Pyranine 10μM; (B) AAPH 5mM, Pyranine 20μM; (C) AAPH 10mM, Pyranine 10μM; (D) AAPH 10mM, Pyranine 20μM.

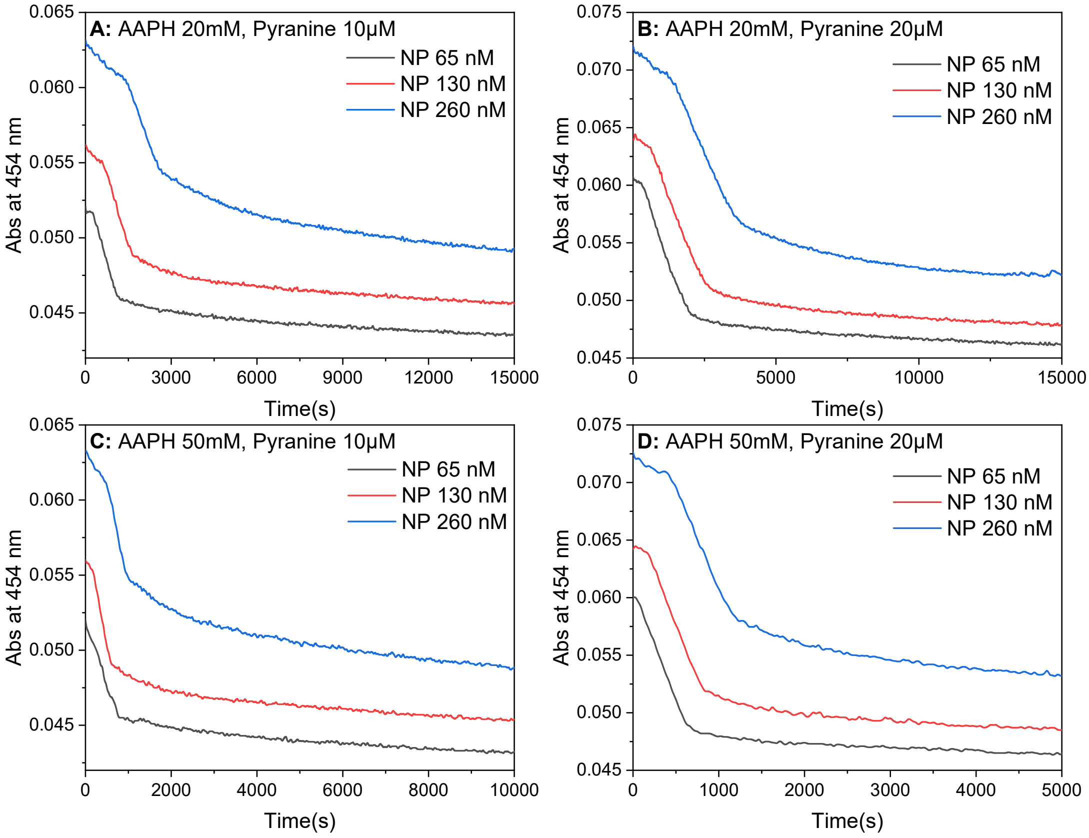

Figure S16. Pyranine consumption caused by radical oxidation triggered by AAPH at 37 °C under the protection of Ce_xO_y-PEG-long. Experiments measured at different pyranine and AAPH concentrations separately: (A) AAPH 20mM, Pyranine 10μM; (B) AAPH 20mM, Pyranine 20μM; (C) AAPH 50mM, Pyranine 10μM; (D) AAPH 50mM, Pyranine 20μM.

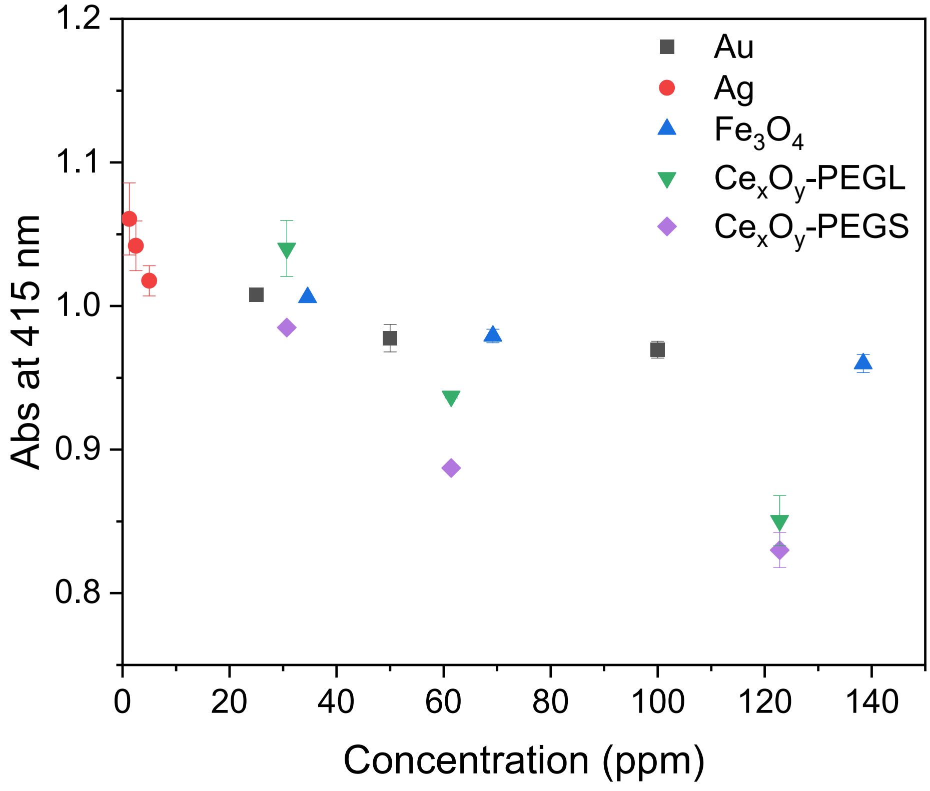

Figure S17. Absorbance of $ABTS^{\cdot+}$ at 415 nm measured 10 mins after the injection of H_2O_2/myoglobin solution under the protection of five types of nanoparticles. Each nanoparticle measured was duplicated with three different concentrations.

Table S1. Physical properties (diameters, densities, and concentrations) of nanoparticles used in TEAC measurements.

Nanoparticles	Surface Coating	Diameter (nm)	Density (g/cm^3)	Metal conc (ppm)	NP core conc (ppm)	Metal (%)	NP Conc (nM)
Au	PEG-SH (10K)	17.7 ± 3.3	19.3	84	84.0	100%	2.5
Ag	PEG-SH (10K)	4.5 ± 0.6	10.49	15	15.0	100%	49.8
Fe_3O_4	PAA (6K)	40.0 ± 3.0	5.17[a]	50	69.2	72.3%	66.3[b]
Ce_xO_y-PEGL	PEGL[c]	4.7 ± 1.0	7.22	10	12.3	81.4%	42.3
Ce_xO_y-PEGS	PEGS[d]	4.7 ± 1.0	7.22	10	12.3	81.4%	42.3

[a] Density of Fe_3O_4 cluster has an estimated value of ~2.5g/cm^3. [b] Concentration of Fe_3O_4 suggests the concentration of particles on clusters. [c] PEGL or PEG-long represents nitrodopamine-PEG (10K). [d] PEGS or PEG-short represents PEG (2K)-PE (1,2-dioleoyl-sn-glycero-3-phosphoethanolamine-N-PEG(2K)).

Table S2. TEAC of five types of nanoparticles calculated by the area under the curve (AUC) in ORAC assays using fluorescein, pyranine, pyrogallol red, and rhodamine B as probes.

		TEAC (µmol Trolox/µmol NP)			
**Assays**	**NPs**	**Before Correction**	**std**	**After Correction**	**std**
Fluorescein	Au	1323.0	275.8	1212.1	207.3
	Ag	212.9	27.3	190.5	31.0
	Fe_3O_4	20.6	6.3	4.3	0.7
	Ce_xO_y-PEGL	57.0	6.4	45.3	4.0
	Ce_xO_y-PEGS	17.3	4.4	13.2	0.0
Pyranine	Au	57012.1	8650.7	16017.2	6863.3
	Ag	4347.1	1461.1	1428.0	182.5
	Fe_3O_4	120.3	17.5	--	--
	Ce_xO_y-PEGL	381.5	66.8	153.3	36.3
	Ce_xO_y-PEGS	1.2	3.8	10.7	5.0
Rhodamine B	Au	558409.2	45232.2	33428.1	4245.4
	Ag	25711.1	5084.7	2650.4	143.8
	Fe_3O_4	--	--	--	--
	Ce_xO_y-PEGL	3891.4	325.5	754.8	49.0
	Ce_xO_y-PEGS	866.2	155.7	282.8	30.5
Pyrogallol red	Au	731360.7	46752.4	--	--
	Ag	3942.6	821.6	2468.4	27.8
	Fe_3O_4	369.0	11.8	--	--
	Ce_xO_y-PEGL	56.6	11.6	14.0	4.0
	Ce_xO_y-PEGS	--	--	--	--

Table S3. (Continued) TEAC of five types of nanoparticles calculated by the area under the curve (AUC) in ORAC assays using fluorescein, pyranine, pyrogallol red, and rhodamine B as probes.

TEAC (µmol Trolox/100mg NP)				TEAC (µmol Trolox/m^2 S.A. NP)			
Before Correction	std	After Correction	std	Before Correction	std	After Correction	std
3.9	0.8	3.6	0.6	0.4	0.1	0.4	0.1
68.1	8.7	60.9	9.9	5.3	0.7	4.8	0.8
19.7	6.1	4.1	0.6	0.1	0.0	0.0	0.0
24.1	2.7	19.2	1.7	1.4	0.2	1.1	0.1
7.3	1.9	5.6	0.0	0.4	0.1	0.3	0.0
169.7	25.8	47.7	20.4	19.3	2.9	5.4	2.3
1391.1	467.6	456.9	58.4	109.4	36.8	35.9	4.6
115.2	16.7	--	--	0.4	0.1	0.1	0.0
161.5	28.3	64.9	15.4	9.1	1.6	3.7	0.9
0.5	1.6	4.5	2.1	0.0	0.1	0.3	0.1
1661.9	134.6	99.5	12.6	188.7	15.3	11.3	1.4
8227.6	1627.1	848.1	46.0	646.8	127.9	66.7	3.6
--	--	--	--	2.2	0.4	0.5	0.1
1647.8	137.8	319.6	20.7	93.2	7.8	18.1	1.2
366.8	65.9	119.7	12.9	20.7	3.7	6.8	0.7
2176.7	139.1	--	--	247.1	15.8	--	--
1261.6	262.9	789.9	8.9	99.2	20.7	62.1	0.7
353.5	11.3	--	--	0.3	0.1	0.2	0.0
24.0	4.9	5.9	1.7	1.4	0.3	0.3	0.1
--	--	--	--	--	--	--	--

Table S4. TEAC of five types of nanoparticles calculated by the half-life time ($t_{1/2}$) in ORAC assays using fluorescein, pyranine, pyrogallol red, and rhodamine B as probes.

Assays	NPs	TEAC (μmol Trolox/μmol NP)			
		Before Correction	std	After Correction	std
Fluorescein	Au	--	--	--	--
	Ag	692.1	83.4	781.3	29.9
	Fe_3O_4	--	--	--	--
	Ce_xO_y-PEGL	120.3	0.9	178.2	1.1
	Ce_xO_y-PEGS	17.9	0.2	31.8	0.1
Pyranine	Au	--	--	--	--
	Ag	188.2	55.1	--	--
	Fe_3O_4	43.5	31.0	--	--
	Ce_xO_y-PEGL	34.4	2.3	135.6	26.2
	Ce_xO_y-PEGS	7.1	2.2	60.5	0.0
Rhodamine B	Au	--	--	--	--
	Ag	3.9	1.5	6.5	2.8
	Fe_3O_4	--	--	--	--
	Ce_xO_y-PEGL	4.2	0.2	4.4	0.5
	Ce_xO_y-PEGS	1.1	0.1	1.1	0.2
Pyrogallol Red	Au	133680.6	40547.1	--	--
	Ag	4015.0	1034.4	3779.7	76.0
	Fe_3O_4	63.7	14.0	--	--
	Ce_xO_y-PEGL	8.7	6.6	--	--
	Ce_xO_y-PEGS	15.9	7.4	--	--

Table S5. (Continued) TEAC of five types of nanoparticles calculated by the half-life time ($t_{1/2}$) in ORAC assays using fluorescein, pyranine, pyrogallol red, and rhodamine B as probes.

TEAC (µmol Trolox/100mg NP)				TEAC (µmol Trolox/m² S.A. NP)			
Before Correction	std	After Correction	std	Before Correction	std	After Correction	std
--	--	--	--	--	--	--	--
221.5	26.7	250.0	9.6	17.4	2.1	19.7	0.8
--	--	--	--	0.1	0.0	0.1	0.0
50.9	0.4	75.4	0.5	2.9	0.0	4.3	0.0
7.6	0.1	13.5	0.0	0.4	0.0	0.8	0.0
--	--	--	--	--	--	--	--
60.2	17.6	292.9	49.1	4.7	1.4	23.0	3.9
41.6	29.7	--	--	0.0	0.0	0.1	0.0
14.6	1.0	57.4	11.1	0.8	0.1	3.3	0.6
3.0	0.9	25.6	0.0	0.2	0.1	1.5	0.0
--	--	--	--	--	--	--	--
1.2	0.5	2.1	0.9	0.1	0.0	0.2	0.1
--	--	--	--	--	--	--	--
1.8	0.1	1.9	0.2	0.1	0.0	0.1	0.0
0.5	0.1	0.5	0.1	0.0	0.0	0.0	0.0
397.9	120.7	--	--	45.2	13.7	--	--
1284.8	331.0	1209.5	24.3	101.0	26.0	95.1	1.9
61.0	13.4	--	--	0.4	0.1	0.3	0.0
3.7	2.8	--	--	0.2	0.2	--	--
6.7	3.2	--	--	0.4	0.2	--	--
